# Neurochallenges in smart cities: state-of-the-art, perspectives, and research directions

**DOI:** 10.3389/fnins.2024.1279668

**Published:** 2024-12-18

**Authors:** Begüm Özkaynak, Necati Aras, İrem Daloğlu Çetinkaya, Cem Ersoy, Özlem Durmaz İncel, Mutlu Koca, İrem Nalça, Turgut Tüzün Onay, Sinan Öncü, Berivan Ülger Vatansever, Eda Yücesoy, Can A. Yücesoy

**Affiliations:** ^1^Department of Economics, Boğaziçi University, Istanbul, Türkiye; ^2^Department of Industrial Engineering, Boğaziçi University, Istanbul, Türkiye; ^3^The Institute of Environmental Sciences, Boğaziçi University, Istanbul, Türkiye; ^4^Department of Computer Engineering, Boğaziçi University, Istanbul, Türkiye; ^5^Department of Electrical and Electronics, Boğaziçi University, Istanbul, Türkiye; ^6^Department of Mechanical Engineering, Boğaziçi University, Istanbul, Türkiye; ^7^The Institute of Biomedical Engineering, Boğaziçi University, Istanbul, Türkiye; ^8^Department of Urban and Regional Planning, Istanbul Technical University, Istanbul, Türkiye

**Keywords:** smart city development, adaptive resource use, human-centered technology, neurourbanism, hybrid intelligence, human wellbeing, resilience and sustainability

## Abstract

Smart city development is a complex, transdisciplinary challenge that requires adaptive resource use and context-aware decision-making practices to enhance human functionality and capabilities while respecting societal and environmental rights, and ethics. There is an urgent need for action in cities, particularly to (i) enhance the health and wellbeing of urban residents while ensuring inclusivity in urban development (e.g., through the intelligent design of public spaces, mobility, and transportation) and (ii) improve resilience and sustainability (e.g., through better disaster management, planning of city logistics, and waste management). This paper aims to explore how neuroscientific and neurotechnological solutions can contribute to the development of smart cities, as experts in various fields underline that real-time sensing designs and control algorithms inspired by the brain could help build and plan urban systems that are healthy, safe, inclusive, and resilient. Motivated by the potential interplay between societal challenges and these emerging technologies, we provide an overview of state-of-the-art research through a bibliometric analysis of neurochallenges within the context of smart cities using terms and data extracted from the Scopus database between 2018 and 2022. The results indicate that smart city research remains fragmented and technology-driven, relying heavily on internet of things (IoT) and artificial intelligence (AI)-based technologies. Mostly, it also lacks careful integration and adoption tailored to societal goals and human-centric concerns. In this context, the article explores key research streams and discusses how to create new synergies and complementarities in the challenge-technology intersection. We conclude that realizing the vision of smart cities at the nexus of neuroscience, technology, urban space, and society requires more than just technological progress. Integrating the human dimension alongside various technological tools and systems is crucial. This necessitates better interdisciplinary collaboration and co-production of knowledge toward a hybrid intelligence, where synergies of education and research, technological innovation, and societal innovation are genuinely built. We hope the insights from this analysis will help orient neurotechnological interventions on urban living and ensure they are more responsive to societal and environmental challenges as well as to legal and ethical concerns.

## 1 Introduction

Smart city development is a complex, multidisciplinary problem that requires adaptive resource use and context-aware decision-making practices to enhance human functioning and capabilities while respecting societal and environmental rights and ethics (March, 2019; Townsend, 2013). Although there is no universally accepted definition of smart cities, a broad and inclusive framing of the concept would link and localize various UN Sustainable Development Goals (SDGs): Goal 3 (good health and wellbeing), Goal 4 (quality education and lifelong learning), Goal 7 (affordable and clean energy), Goal 9 (industry, innovation, and infrastructure), Goal 10 (reduced inequalities), Goal 11 (sustainable cities and communities), Goal 13 (climate action), Goal 16 (peace, justice, and strong institutions). To achieve these goals, urban areas must take urgent actions to (a) enhance the health and wellbeing of city residents while ensuring inclusivity in urban life (e.g., through the intelligent design of public spaces, mobility, and transportation) and (b) ensure that urban development and planning contribute positively to resilience and sustainability (e.g., through better disaster management, planning of city logistics, and waste management).

Indeed, the concept of smart cities is also related to the notion of Society 5.0, which was proposed in Japan to form a sustainable societal environment and enhance residents' comfort with enriched technological opportunities, where physical space and cyberspace are integrated in a balanced way through technological advancements such as the Internet of Things (IoT), blockchains, edge computing, and machine learning algorithms (Kiruthika et al., 2024). Another term used for Society 5.0 is human-centric super-smart society. This concept has also impacted Industry 4.0, which primarily focuses on technological advances to form smart infrastructures such as smart transportation, smart buildings, smart factories, and smart healthcare, transforming it into Industry 5.0, where the wellbeing of humans is essential by respecting their working conditions (Coronado et al., 2022). In other words, all the smart infrastructures are established with a human-centric focus, paying attention to human-machine interaction and interfaces. This, in turn, brings the topics of societal values and human wellbeing issues to the forefront, in addition to the economic considerations and efficiency issues of Industry 4.0 (Alter, 2020). It has been well understood that significant technological advances in areas such as transportation, healthcare, communication, education, and manufacturing are insufficient and ineffective for sustainable development without considering human factors. Within the scope of smart cities, it is crucial to integrate the human dimension alongside various technological tools and systems such as IoT, big data, and machine learning algorithms (Komninos and Kakderi, 2019). This necessity brings neurotechnology into play.

In recent years, experts across various fields have acknowledged the significant potential of real-time sensing designs and neuro-inspired control algorithms for local interventions, thereby supporting the development and planning of urban systems that are healthy, inclusive, safe, and resilient. Ancora et al. (2022) highlight substantial advancements in our understanding of brain functioning, with neuroscience expanding its applications across various domains, such as marketing, economics, decision sciences, and educational sciences. This expansion, aided by technological advancements such as wearable devices and software applications, has also given rise to a novel field known as “neurourbanism” which integrates theoretical perspectives and analytical methods inspired by the brain. The aim is to deepen insights into human needs, behaviors, and decision-making processes in urban environments, ultimately enhancing service design and implementation throughout cities in a context-aware manner (Makanadar, 2024).

The study by Gu et al. (2024), for instance, investigates the impact of sidewalk ground murals, focusing on color (warm, cool, or achromatic) and pattern (rectilinear or curvilinear), on mood states and perceived restorativeness, especially under stress. Using a 3 × 2 × 2 mixed design, 112 students were divided into stressed and non-stressed groups and assessed across six mural designs and one uncolored condition. Findings indicate that ground murals enhance mood and perceived restorativeness compared to uncolored sidewalks. Cool colors were most effective in promoting restorativeness, especially for stressed individuals, while warm colors reduced relaxation, and achromatic colors decreased energetic arousal and were least restorative. Pattern type did not enhance mood but curvilinear patterns were perceived as more restorative than rectilinear patterns. The study supports urban design strategies that incorporate systematic use of color and pattern in ground murals to improve mental health. For example, two Superblock initiatives in Barcelona utilized ground murals to create a more inviting environment for pedestrians and cyclists, making the neighborhood more engaging and suitable for community activities (Archdaily, 2020).

Similarly, Mikuni et al. (2024) are interested in whether artistic interventions in urban street environments enhance wellbeing and how these improvements relate to aesthetic evaluations of the environment. After examining three hypotheses, the authors find out that engaging with artistic installations in urban street settings alleviates subjective experiences of anxiety, stress, and negative emotions. Negami et al. (2018) examine how urban design elements such as colorful crosswalks and greenery impact mental wellbeing, sociability, and environmental stewardship. Participants in Vancouver's West End evaluated six sites, three with urban interventions and three without, using a smartphone app to express their emotional reactions. Sites with greenery and colorful designs were linked to higher happiness, trust, and attraction. The findings suggest that urban design changes can enhance city residents' wellbeing and sociability.

In another study, Dimitrov-Discher et al. (2022) assess the impact of green space, air pollution, and noise pollution at the place of residence on neurofunctional activation during social stress. Based on fMRI data from 42 participants, the study revealed that exposure to green spaces correlates with increased parietal and insular activation under stress. In contrast, air pollution is related to diminished activation in these brain regions. These outcomes could provide valuable insights for upcoming research in the developing discipline of “neurourbanism” and highlight the critical role of environmental factors in urban design. The findings suggest that simple urban design changes can enhance city residents' wellbeing and sociability. Neurotechnology, in this context, by interacting with the human brain and nervous system, enables technological systems to become more aligned with human needs. Consequently, this integration has the potential to allow smart cities to achieve both efficiency and human-centricity.

**Figure 1:**
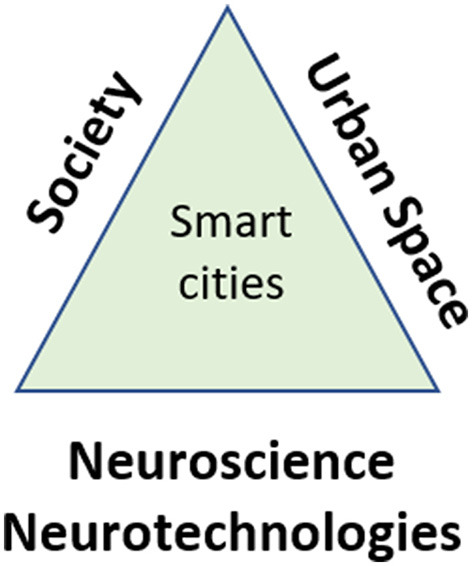
Smart cities as hybrid intelligence in the nexus of neuroscience/technologies, urban space, society.

In the context of smart cities, “neurochallenges” involve using principles of cognition and neuroscience to comprehend and anticipate human behavior and requirements within urban environments. Appropriate solutions are then devised and implemented utilizing neurotechnologies to enhance the quality of life and human wellbeing. This smart city vision, depicted in Figure 1, positions itself at the nexus of neuroscience and technology, urban space, and societal interactions. Realizing this vision requires co-producing knowledge toward a hybrid intelligence, whereby education and research, technological innovation, and societal innovation go hand in hand, addressing key focus areas in line with the SDGs.

It goes without saying that, “going smart” raises significant ethical and privacy concerns that must be carefully managed. Therefore, implementing this vision mandates an interdisciplinary and integrative approach to address the critical issues and concerns of urban development and planning by allowing the consistent and coherent communication of multiple codes and perspectives (Kourtit and Nijkamp, 2012; Castells, 2000) and strong collaboration among governments, industry, academia, and civil society organizations (Breuer et al., 2019). Again, the ultimate goal is not just to improve effectiveness but to ensure that technological advances serve human wellbeing and promote healthier and more sustainable urban planning and design purposes (Pykett et al., 2020).

Against this background and motivated by the potential interplay between societal challenges and emerging technologies, in this paper, we undertake a bibliometric analysis of the literature on smart cities through the lens of neuroscience and neurotechnology. Using data from 2018 to 2022 extracted from the Scopus database, based on a list of terms and themes identified within the scientific community, we provide a comprehensive overview of the research landscape around smart cities to address the following four research gaps: (1) mapping the knowledge structure in the literature around smart cities and exploring emerging topics in neuroscience and neurotechnology as they apply to smart cities; (2) evaluating to what extent technological solutions and advances effectively address societal and environmental challenges in the 21st century; (3) discussing, using key research streams, how to better create synergies and complementarities to contribute to the overarching goals of health, inclusivity, safety, and resilience in urban development; (4) providing insights for strategic planning and future research directions to policymakers, funding agencies, and institutions.

**Figure 2:**
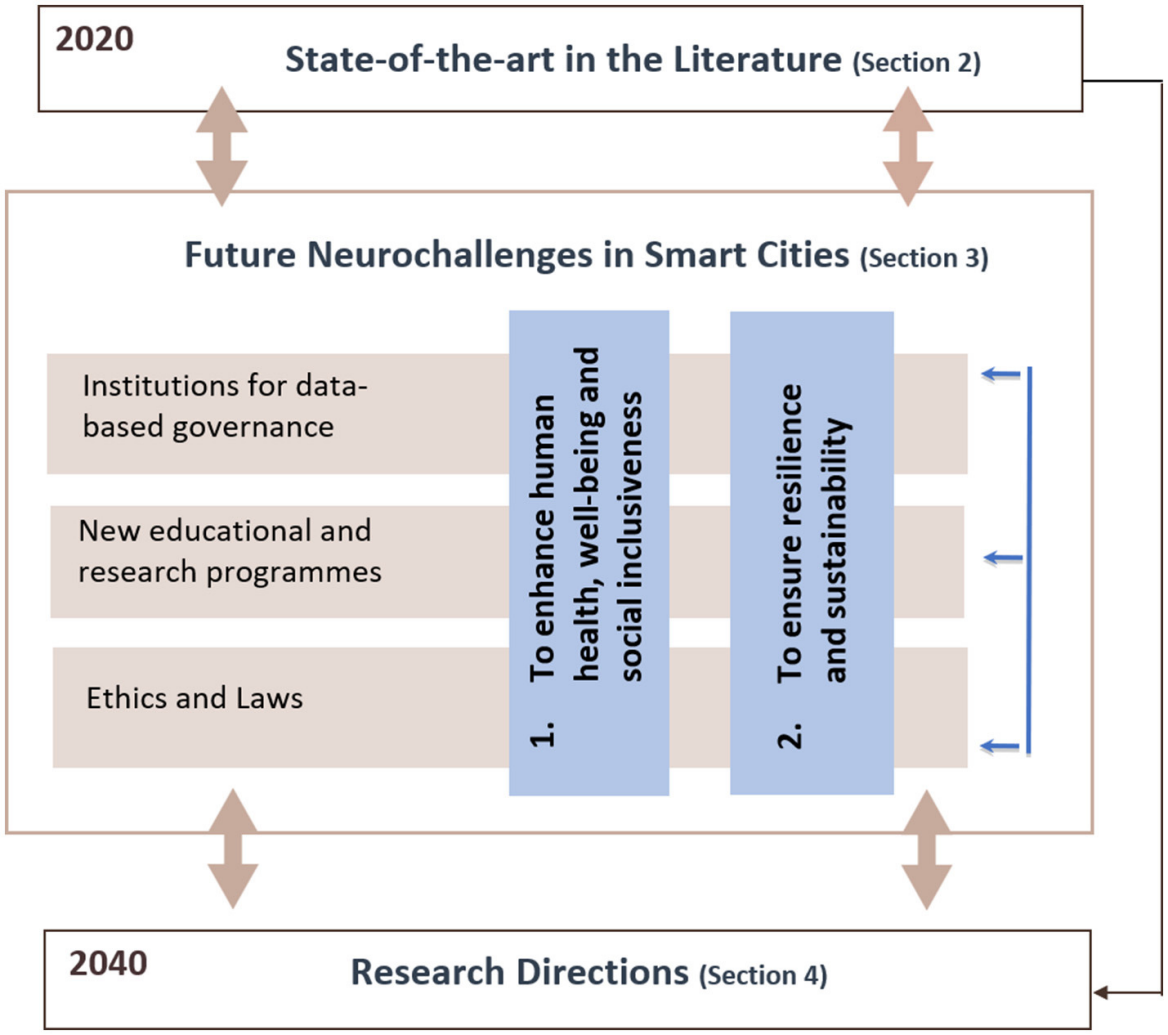
Purposive areas for neuroscience and neurotechnologies in smart cities.

While some earlier studies offer reviews of smart city research, they do not explicitly tackle the questions and issues discussed here. Zhao et al. (2021) provide a systematic review of highly cited smart city literature based on the analysis of 191 publications. Allam and Dhunny (2019) focus in their review on the opportunities and challenges of big data and artificial intelligence and discuss how to govern them so that they are integrated into the urban societal fabric. Ruhlandt (2018) analyzes the relevant body of literature on smart city governance to better understand its different components and the metrics used to measure outcomes. Mora et al. (2019) examine a group of literature reviews and studies to highlight the lack of consensus on selecting an approach to effectively manage smart city development, arguing that this undermines smart city practice and the potential for urban sustainability. Özdemir et al. (2019) conduct a qualitative review of smart city literature, concluding that rapid ICT development has exacerbated developmental gaps within and between urban areas. They emphasize that the social dimension is often neglected, underlining the need for smart city strategies to address both socioeconomic and quality of life dimensions. In this context, Figure 2 illustrates the scope and structure of this paper, emphasizing the unique contributions and perspectives explored in this study.

In Section 2, we present the state-of-the-art in the literature by offering a bibliometric analysis of neurochallenges within the context of smart cities. After explaining the methodology and describing the data sources used for the bibliometric analysis, we first present our findings and insights, initially on a broad basis, and then focus on four specific sub-disciplines (computer science, engineering, social sciences, and environmental sciences). Section 3 illustrates the potential use and practical applications of neurotechnological solutions in the context of smart cities and aims to discuss how better challenge-technology synergies might be built. In particular, we consider the implications of neurotechnology in two purposive areas aligned with the SDGs (represented in blue color in Figure 2), which are (i) human health, wellbeing, and social inclusiveness and (ii) resilience and sustainability. For the former, we focus on neurodesign, followed by a discussion on mobility and access to transportation. For the latter, we investigate disaster and waste management for ecosystem resilience and health.

Then, Section 4 emphasizes some specific areas that require the attention of academic and policy circles, particularly emphasizing three interrelated concerns (represented in pink color in Figure 2) that must be concurrently addressed from a systemic standpoint to ensure that societal goals and technologies are not overlooked or sidelined. These concerns involve acting and establishing (a) institutions promoting data-driven governance, transparency, and accountability to support public decision-making processes in these two purposive areas, necessitating collaboration among multiple stakeholders (b) new educational and research initiatives like NeurotechEU, the European University of Brain and Technology, which represents an alliance of nine European universities focused on neurotechnology (c) regulatory frameworks and ethical codes to ensure the responsible development and application of these technologies. Lastly, Section 5 provides insights on future research directions and concludes the paper.

## 2 A bibliometric analysis of neurochallenges within the context of smart cities

In this section, we introduce the details of our research, presenting the methodology and initial outcomes. We first explain how we conducted the bibliometric analysis and the sources from which we extracted data. This is followed by the presentation of the co-word analysis conducted on all selected articles. We then shift to a more discipline-focused perspective, performing a co-word analysis with a particular disciplinary emphasis. Finally, we present the critical insights derived from our bibliometric analysis.

### 2.1 Methodology and data sources

The main goal of our bibliometric study is to reveal the intellectual structure and important themes in the field of research. To achieve this, we initially generated a draft list of terms and refined and augmented them into a finalized list through consultation with experts, as can be seen in Table 1. This represents the first step in our bibliometric methodology. In the second step, we ensured the relevance of our bibliometric analysis to smart city research by narrowing our search to those papers where the “smart cities/city” keyword appeared, along with terms and themes representing Neuroscience and Neurotechnology in relation to Smart Cities (listed in Table 1) or terms and themes characterizing a Smart City concept (listed in Table 2). When one searches these lists with the concept of a “smart city,” the evolution of the idea becomes apparent. It shifts from being primarily about technology to focusing on people, eventually embracing inclusive and participatory governance.

**Table 1:** Terms and themes representing neuroscience and neurotechnology in relation to smart cities.

• Neuroaesthetics	• Neuroarchitecture
• Neurolaw	• Neurourbanization
• Neurophilosophy	• Neuroart
• Neuroethics	• Neurodesign
• Mental health	

**Table 2:** Terms and themes characterizing smart city concept.

• Mobility	• Bottom-up governance
• Access to public transportation	• Data-based governance
• Micro-mobility problems	• Evidence-based decision-making
• Socially inclusiveness	• Societal innovation
• Care solutions	• Open-data platforms
• Physical and social landscapes	• Technology as commons
• Comfort and wellbeing	• Technological literacy
• Harmony	• Dissemination
• Smart design of public space	• Citizen science
• Frugal technologies	• Waste management
• Resilience	• Classification for recycling
• Real-time resilience	• Recycling of plastics
• Adaptation	• Water management
• Human-centered design	• Sustainability
• Disaster management	• Resource-aware planning
• Nudges	• Interaction of autonomous vehicles

In the third step, we searched the Scopus database, preferring it over the Web of Science due to its greater number of indexed journals. Using the query function, we looked for articles containing these terms in the article title, abstract, or keywords, covering all research institutions for the last 5 years, from 2018 to 2022. For this step, following typical literature review practices, we restricted our search to peer-reviewed journal articles and conference publications, thereby excluding gray literature and books. Furthermore, we only included articles in English. Our search criteria and terms yielded 27,346 peer-reviewed manuscripts. In the final stage, to streamline the data in our bibliometric analysis, we also created a thesaurus and combined or recoded some words following bibliometric analysis guidelines (Donthu et al., 2021), addressing variations in term usage across different publications, such as the singular or plural forms or the use of acronyms. For instance, we made no distinction between “smart city” and “cities” or between “mobile crowdsensing” and “mcs”. This enabled us to generate a reliable list of author keywords in our dataset.

**Figure 3:**
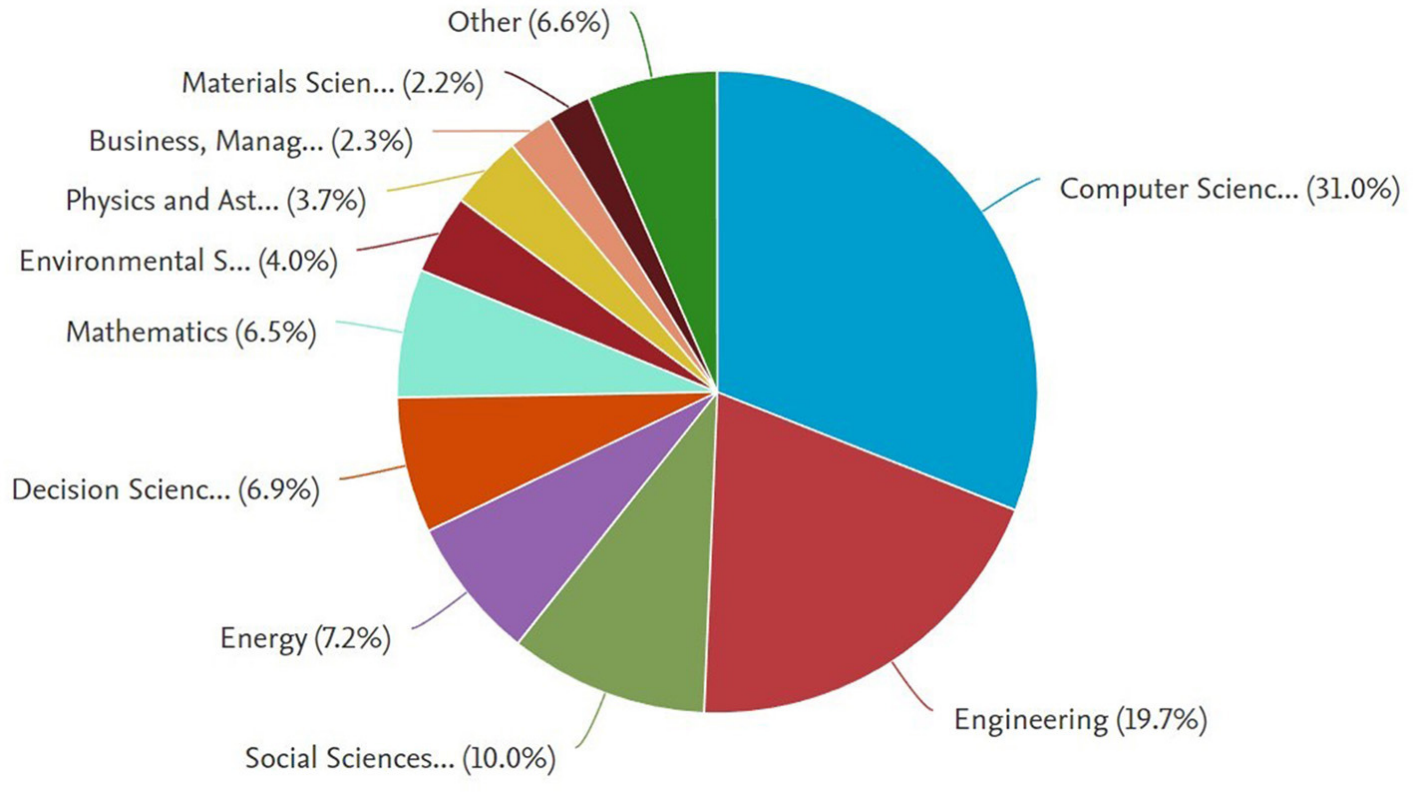
Bibliometric analysis: share of various disciplines (2018–2022).

We also analyzed the disciplinary foundations of the bibliometric data at our disposal. Figure 3 illustrates the diversity of fields covered by the literature, each highlighting different aspects of smart cities and reflecting the varied goals and priorities that stakeholders may have.

The most prominent subject area is computer science, which contains 19,910 documents, accounting for 31.0% of the overall documents. Engineering ranks second with 11,893 documents, constituting 19.7% of the overall number of documents. Therefore, half of the global research in Smart Cities is highly technical. The social sciences discipline ranks third with 6,138 documents (10% of documents). Due to its high relevance, we also studied environmental sciences, which contains 2,433 documents. This distribution signals opportunities for dialogue and collaboration between the dominant disciplines and those with smaller representations. Neuroscience does not appear to be a field dominating the scene presently (appearing within the group of subject areas referred to as Others). Still, it might become prominent, given its potential for new collaborations with other disciplines.

### 2.2 Co-word analysis for all articles selected

**Figure 4:**
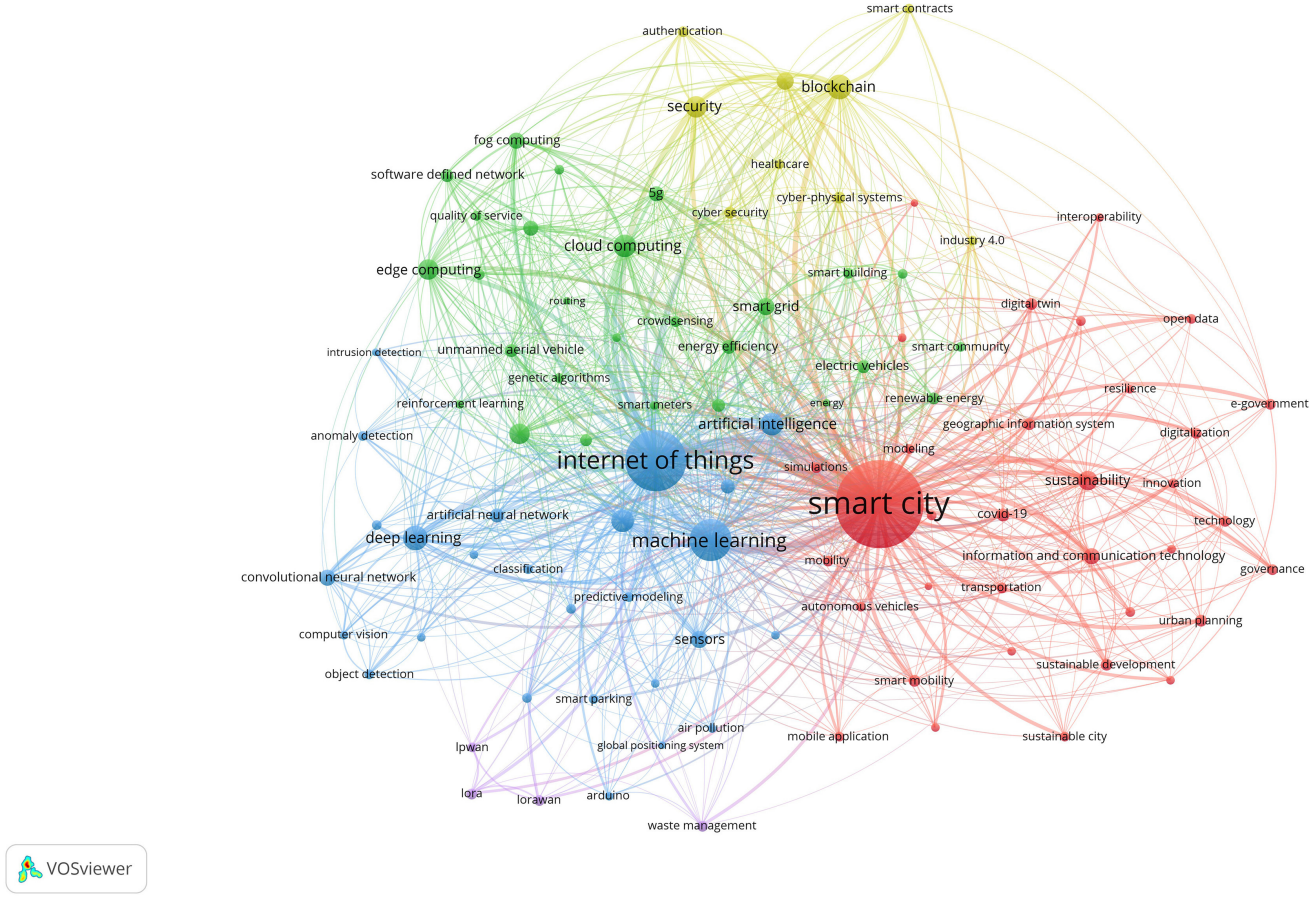
Co-word network visualization (2018–2022).

We conducted a co-word analysis, also known as “author keyword co-occurrence”, on the selected articles in the VOSviewer software. This method identifies keywords that frequently appear together in the content of the articles, indicating thematic relationships. Figure 4 presents one such bibliometric analysis result, a general one conducted without disciplinary filtering. While generating images for different periods is always possible, the image below represents the last 5 years, from 2018 to 2022. We used the VOSviewer clustering algorithm (with the counting method fractional counting and the normalization method based on association strength), which allowed us to generate a number of clusters based on topics. In our analysis, it seemed adequately meaningful to interpret the thematic groups within five clusters.

Each node in this network represents an author-defined keyword, such as “smart city,” “internet of things,” “machine learning,” or “urban planning.” The size of each node represents the frequency of the keyword occurrences. As the node size increases, it indicates higher keyword frequency. Links between nodes imply that corresponding keywords co-occur within articles, with the link thickness representing the strength of co-occurrence. Each color represents a distinct thematic cluster. The nodes and links within a cluster help explain the range of topics (nodes) covered by that theme (cluster) and how those topics (nodes) are interconnected (links) (Donthu et al., 2021). To fully interpret the results of the bibliometric analysis, our team convened to discuss the interpretation of each thematic cluster and the significance of the keywords within publications in that cluster. The following are some preliminary observations drawn from these results.

The red cluster in Figure 4 focuses primarily on sustainability issues, exploring strategies for more sustainable urban development, planning, and governance, which are key driving forces in existing smart city literature (Caragliu et al., 2011). This includes leveraging innovative technologies such as e-government, geographic information systems, and information and communication technologies (ICT) to manage and operate a city more effectively and improve the quality of life. The green cluster centers around using specific digital technologies for addressing particular problems, such as energy consumption and efficiency, smart grids, smart meters, electric vehicles, and edge computing. The blue cluster highlights the “smart city” concept, emphasizing techniques from areas like “machine learning” and “artificial intelligence.” The yellow cluster addresses privacy and security concerns, touching on “authentication” and “smart contracts.” Lastly, the purple cluster may be considered a niche theme, delving into network and gateway protocols like LoraWAN and LPWAN (see Supplementary Table SA for a detailed breakdown of the keywords in each of the five clusters, and their frequency of occurrence).

### 2.3 Co-word analysis with a disciplinary focus

In recognition of the integral link between smart cities and the vision of context-aware decision-making, we also aimed at establishing stronger connections to specific SDGs, in particular focusing on SDG3 (good health and wellbeing), SG7 (affordable and clean energy), SDG9 (industry, innovation, and infrastructure), SDG10 (reduced inequalities), SDG11 (sustainable cities and communities), SDG13 (action on climate change) and SDG16 (peace, justice, strong institutions). To this end, we narrowed our search to papers within four primary disciplines: computer science (relevant to SDGs 3, 9, and 11), engineering (relevant to SDGs 7, 9, and 11), social sciences (relevant to SDGs 3, 10, and 16), and environmental sciences (relevant to SDGs 11 and 13).

When we limit the analysis to certain subject areas or subsets of disciplines, more specific thematic groups, or clusters, emerge. This is because each discipline—Computer Science, Engineering, Social Sciences, and Environmental Sciences—has its unique perspective on smart cities. The themes identified from the bibliometric analysis and how they are interpreted in these four disciplines give us which keywords are most frequently used in each field and how many are shared across disciplines. Each figure also provides insight into the meanings of the clusters. While some clusters in these figures seem more goal-oriented or challenge-driven, others are techniques, methods, or technologies. For each subject area, the top 15 keywords were classified as challenges or technologies and presented as a table to support the maps. As such, the most frequently addressed technologies and challenges were underlined. Indeed, this classification highlights the dominant characteristics of the field, as technology-dominant or challenge-driven and, in a way that supports the maps. Understanding key/notable technologies and challenges facilitates discussions about missing links and potential disciplinary partnerships.

#### 2.3.1 Bibliometric analysis for computer science

**Figure 5:**
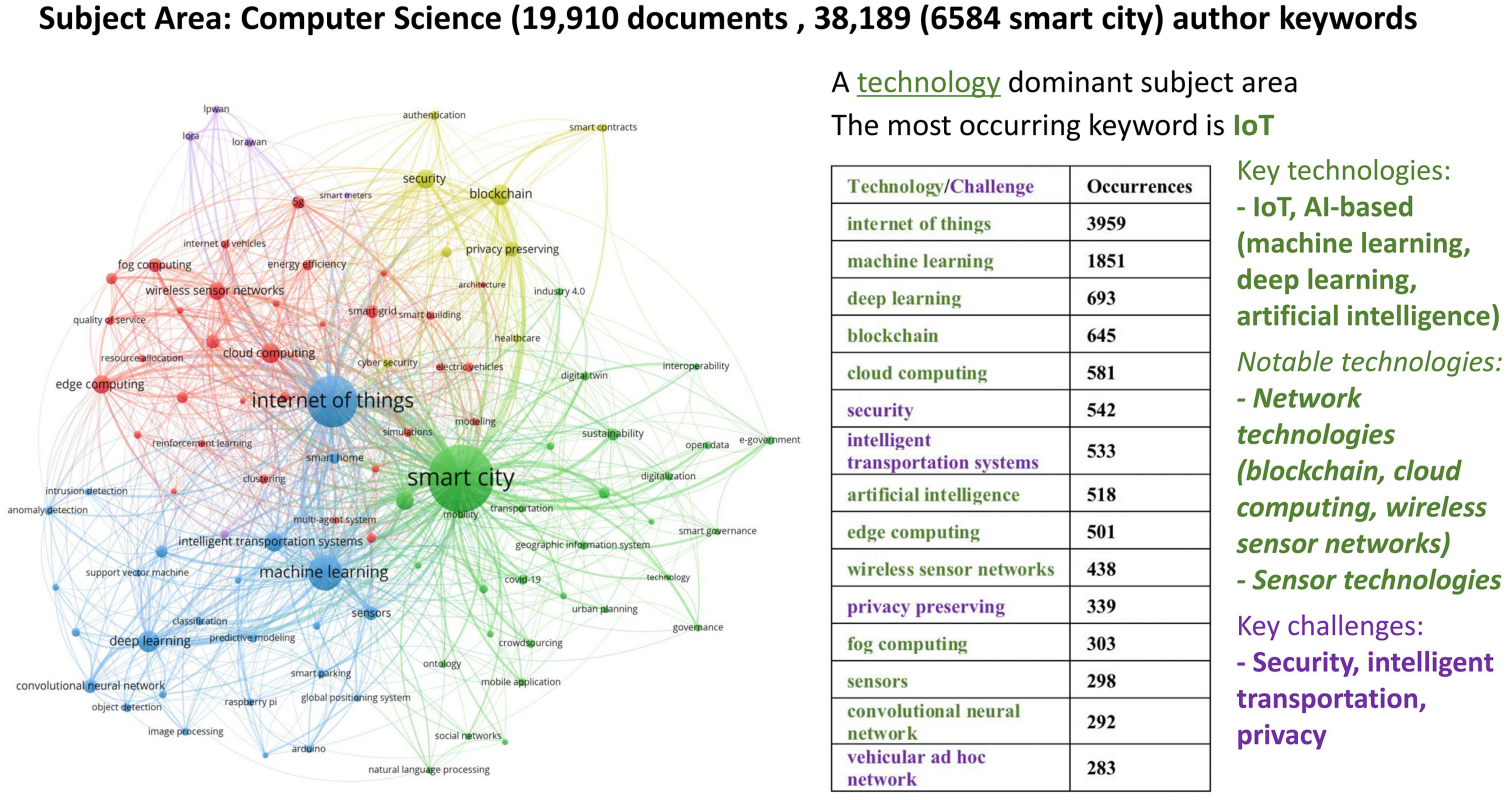
Co-word network visualization with Computer Science focus (2018–2022).

The analysis under computer science was based on 19,910 documents (including peer-reviewed articles and conference papers) and 38,189 author keywords. In Figure 5, we visualize the top 100 keywords with the highest occurrence (the minimum number of author keyword occurrences being 66) under five clusters. This gives us a general overview of the main topics that authors explore in computer science within the context of smart cities. The blue cluster reveals that a significant portion of research in computer science, as expected, relates to the “Internet of things” and techniques such as machine learning, deep learning, image processing, and classification. The red cluster focuses on digital technologies and their infrastructure, encompassing aspects such as cloud computing, wireless sensor networks, edge computing, fog computing, 5G, and the internet of vehicles. The green cluster corresponds to research on smart cities' practical concerns and challenges. Specifically, it represents areas where digital technologies and AI techniques from the blue and red clusters can be applied, such as public transport, smart mobility, information and communication (ICT) technology, e-government, smart governance, smart community, and augmented reality. The yellow cluster emphasizes privacy and cybersecurity concerns. Lastly, the fifth cluster, indicated in purple, centers around a niche topic and explores issues around gateway protocols (see Supplementary Table SB for a detailed breakdown of the keywords in each of the five clusters, along with their frequency of occurrence). Overall, computer science in smart city research seems a technology-dominant subject area, not challenge-driven and not so much integrated into environmental and societal goals.

#### 2.3.2 Bibliometric analysis for engineering

**Figure 6:**
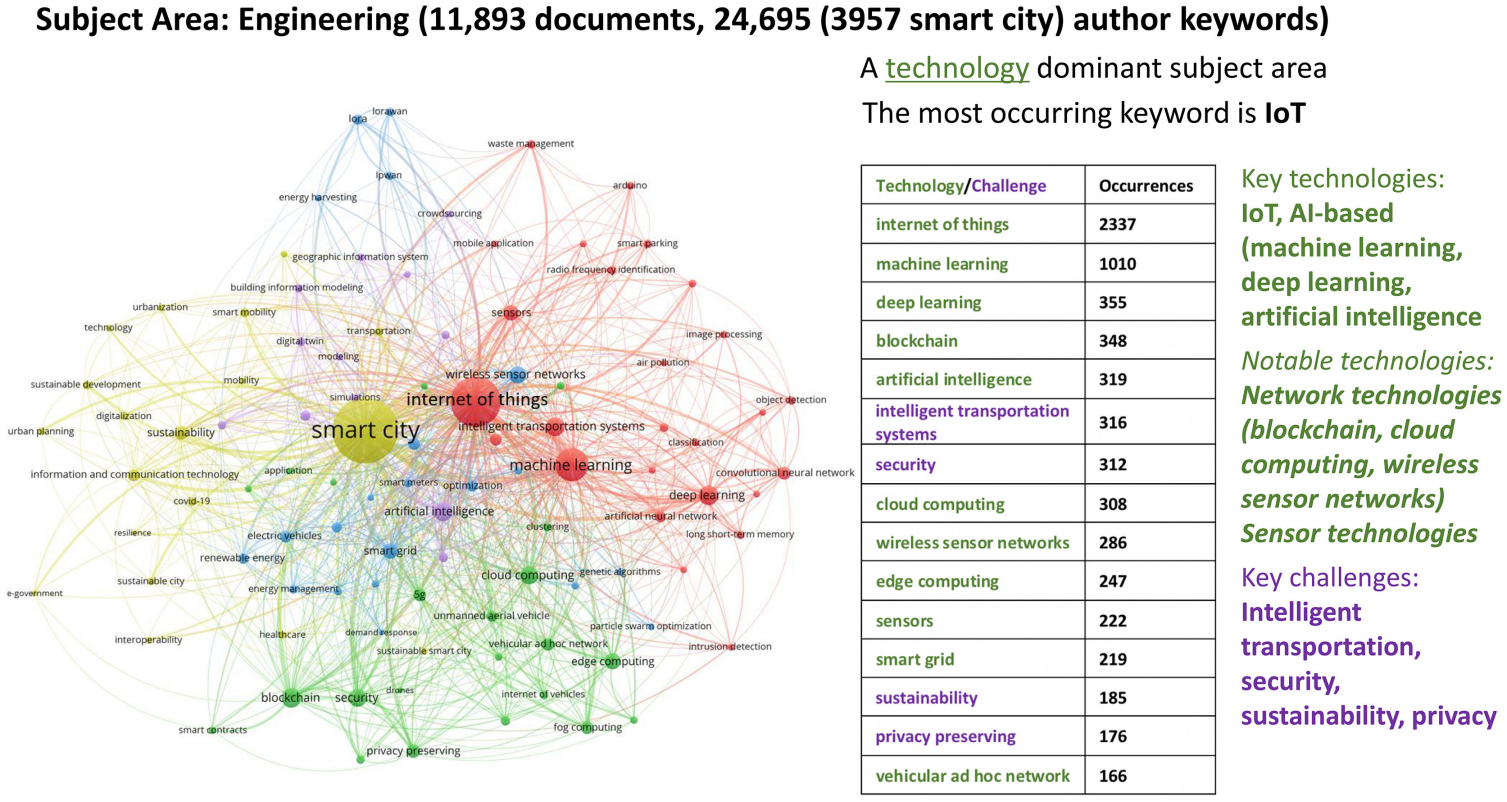
Co-word network visualization with Engineering focus (2018–2022).

The analysis for engineering was conducted on 11,893 documents (peer-reviewed articles and conference papers), and over 24,695 author keywords. In Figure 6, we then visualized the top 100 keywords, with the highest occurrence (the maximum number of keyword occurrences being 3,957 and the minimum being 41) falling into five clusters. The yellow cluster with “smart city” as its central node signifies some primary challenges for engineering encompassing sustainability, urban planning, mobility, resilience, and governance issues. The red cluster centered around the “internet of things” incorporates technologies such as object detection and techniques like deep learning, machine learning, and deep neural networks. These are deployed for specific purposes, such as in smart homes, smart parking, and classification, all represented within the same cluster. Though diverse, the green cluster contains many innovative ICT infrastructures and uses, such as 5G, fog computing, edge computing, blockchain, and the internet of vehicles. The blue cluster focuses on energy-related topics, such as energy efficiency, renewable energy, smart grid, smart meters, and electric vehicles. The purple cluster features a large central node that connects the “artificial intelligence,” to cyber security, and information models and systems (see Supplementary Table SC for a detailed breakdown of the keywords in each of the five clusters, along with their frequency of occurrence). While the engineering subject area in smart city research is also technology-dominant, the relatively frequent occurrence of sustainability keywords underlines its problem-solving-oriented character.

#### 2.3.3 Bibliometric analysis for social sciences

**Figure 7:**
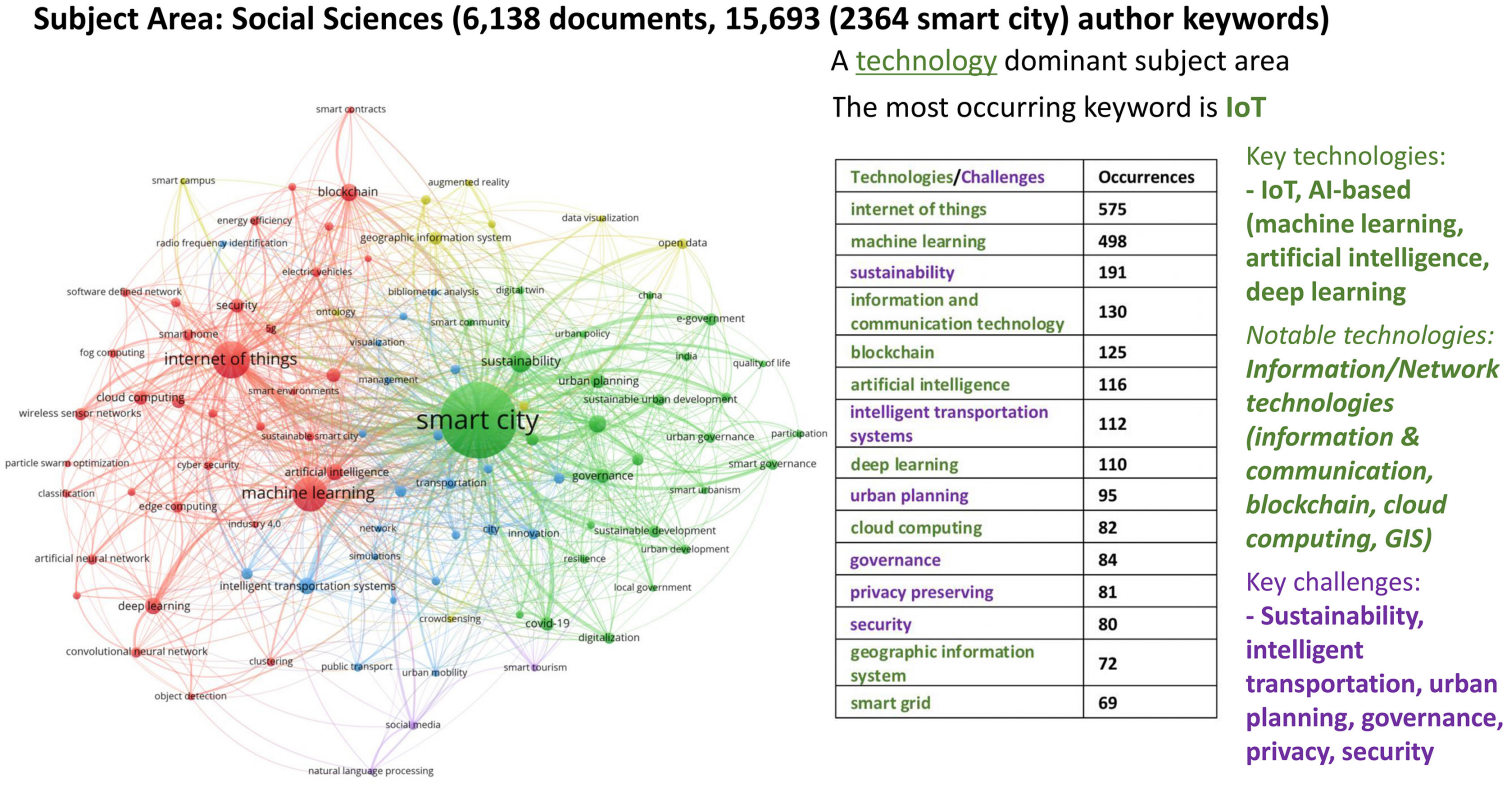
Co-word network visualization with Social Sciences focus (2018–2022).

In social sciences, we analyzed 6138 documents (peer-reviewed articles and conference papers) encompassing 15,693 author keywords. In Figure 7, we visualize the top 100 keywords with the highest occurrence (the maximum number of keyword occurrences being 2,364 and the minimum 21) in five clusters. Three large clusters (red, green, and blue) with many keywords and two relatively smaller groups stand out. The green group considers the core of smart cities to be sustainable urban development, urban planning, governance, participation, and quality of life. These ideas are presumably made workable by connecting them to the other large cluster, the red areas, where techniques such as the internet of things, machine learning, and AI are used. The blue cluster in the social sciences covers issues surrounding intelligent transportation systems linked to smart mobility and public transport. The yellow cluster then examines the uses of technologies such as data visualization, virtual reality, and augmented reality. The purple cluster is small, featuring social media and smart tourism as niche topics (see Supplementary Table SD for a detailed breakdown of the keywords in each of the five clusters, and their frequency of occurrence). In the social sciences, while there is more emphasis on challenges compared to computer science and engineering, the fact that even in this subject area, smart city research emerges as a technology-dominant subject area underlines the need to create better synergies between technologies and challenges.

#### 2.3.4 Bibliometric analysis for environmental sciences

**Figure 8:**
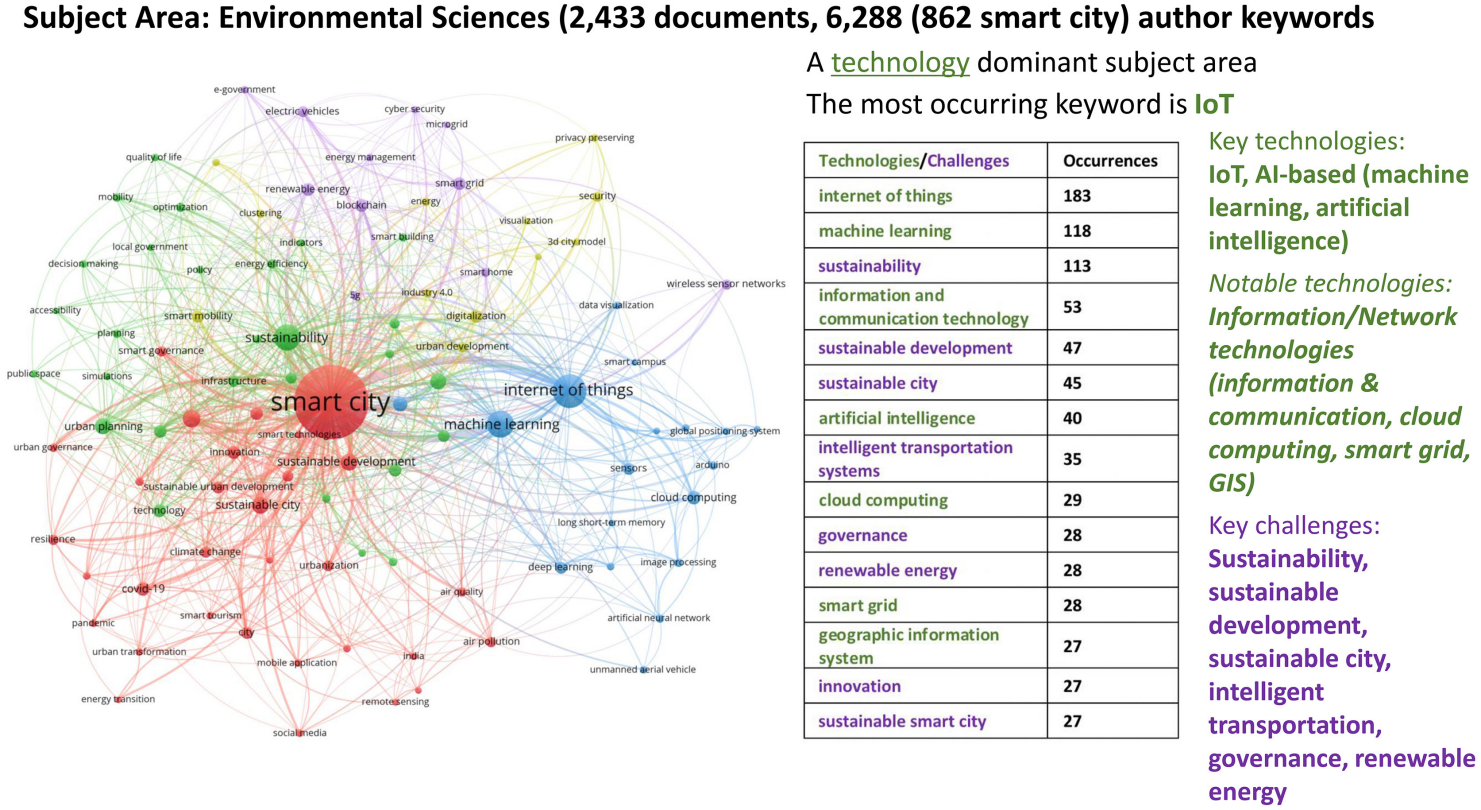
Co-word network visualization with Environmental Sciences focus (2018–2022).

The analysis of 2,433 documents (peer-reviewed articles and conference papers) within environmental sciences gives us 6,288 author keywords. Figure 8 reflects the top 100 keywords with the highest occurrence (the minimum number of keyword occurrences being 8). The red cluster of the network represents challenges such as eco-cities and sustainable urban development from an environmental science perspective. It includes issues such as air quality, climate change, energy transition, urban transformation, and the circular economy. With sustainability and quality of life concerns, the green cluster focuses on planning and decision sciences, where optimization, indicators, and infrastructure policies play a role. The blue cluster is centered mainly around internet of things and machine learning, representing computer science solutions along with sensors, data visualization, and image processing. The purple and yellow clusters are small niche areas around energy and security (see Supplementary Table SE for a detailed breakdown of the keywords in each of the five clusters, along with their frequency of occurrence). While being still technology-dominant, environmental sciences in smart city research are where there exist signs of better synergies and integration between challenges and technologies.

### 2.4 Key insights from the bibliometric analysis

Our analysis presents compelling evidence that smart city development encompasses diverse dimensions, necessitating the application of multiple perspectives to address the societal and environmental challenges in urban settings. Within our bibliometric analysis, numerous author keywords consistently emerged, signifying recurring themes and shared terminologies across disciplines at the intersection of neuroscience, technology, urban space, and society. A clear trend is that certain key technologies dominate smart city research across all subject areas: Internet of Things (IoT), AI-based techniques/technologies (including machine learning, artificial intelligence, and data mining), and information/network technologies (such as wireless sensor networks, cloud computing, edge computing, blockchain, smart grids, GIS, and mobile applications). These technologies stand as humanity's core infrastructures, and they are relevant for smart cities as well and will surely play a fundamental role in shaping urban processes. In contrast, core challenges aligning with SDGs, such as urban planning, sustainability, e-government, climate change, resilience, healthcare, smart mobility and intelligent transport systems, energy efficiency, air and water quality, and smart homes, are discussed less frequently. This disparity indicates a fragmentation in the interface between challenges and technologies. Therefore, creating better synergies at this intersection seems crucial to ensure that technological advancements address urban challenges effectively and in alignment with the SDGs. Naturally, there are variations in core focuses among disciplines, reflecting researchers' and stakeholders' diverse goals and priorities. For example, the computer science perspective predominantly revolves around the role of technological solutions and ICT infrastructures. Technological solutions and problem-solving approaches primarily guide the engineering perspective. The social sciences perspective emphasizes theoretical and conceptual frameworks that shape urban development and address organizational concerns such as e-governance and citizen participation. Yet, the coherence between challenges and technologies in the subject area of social sciences is remarkably and unexpectedly lacking, as, above all, this is also a technology-dominant subject area. Key technologies studied globally in social sciences are identical to those in the technical sciences of computer science and engineering, i.e., IoT and AI-based technologies (machine learning, artificial intelligence, deep learning). The environmental perspective integrates high-tech solutions with green interventions to achieve environmental goals. Nonetheless, a more integrated approach is needed to better align technologies with the pressing societal and environmental challenges of our time.

One potential risk in smart city development is the possibility of incoherent and inadequate implementation or a lack of coordination in addressing common urban challenges. The overall complexity of urban development poses a danger of prioritizing short-term gains through opaque technological solutions. Seeing the big picture across disciplines holds significant value for academics and policymakers, providing them with insights to establish synergies and better complementarities. Indeed, this brings us to the importance of transdisciplinarity, a process that is inherently complex and challenging. It was originally defined as the coordination of all disciplines and interdisciplines within an education/innovation system based on some common objectives to deliver on the purpose of societal continuous self-renewal (Jantsch, 1970). This critical concept and its implications at the interface of science and society will be explored further in Section 4.2.

It is important to note that this analysis is primarily quantitative and focuses on the frequency of keywords, which may not capture the full depth of the research landscape. A more nuanced question arises here: Do the outcomes of the most frequently studied three subject areas actually support the hybrid intelligence-based co-production of knowledge? Or do they reflect a technology-dominant approach conducted by technical experts, with results published in social sciences journals? A qualitative and systematic study that examines the content and context of the documents would complement this analysis and provide a richer understanding. Additionally, considering the temporal aspect of the keywords and documents could offer a more detailed understanding of emerging trends and shifting research priorities over time.

The acknowledgment of noticeable developments and what has been achieved so far, as well as the identification of the critical aspects that have been overlooked but need to be developed enable researchers and policymakers to be aware of potential disciplinary bridges. This comprehension is essential for formulating cohesive and integrated strategies and advancing smart city initiatives in alignment with SDGs.

Building upon these insights, Section 3 of our study presents specific perspectives—from various fields of study—regarding the implications of neurotechnology in smart cities. These perspectives encompass the associated challenges and neurotechnological solutions. Any unresolved and open issues in this context will then be discussed in Section 4.

## 3 Perspectives on implications of neurotechnology in smart cities

The discussion in this section is structured in two parts. The first part highlights specific challenges and corresponding neurotechnologies in smart cities aimed at enhancing human health, wellbeing, and social inclusiveness. The second part discusses, in a similar vein, neurotechnologies that contribute to the resilience and sustainability of smart cities, in particular, through improved disaster management and waste management.

### 3.1 Neurotechnologies in smart cities for human health and wellbeing

#### 3.1.1 Challenges in the intelligent design of public space, mobility, and transportation

Modern urban life is very demanding in terms of economic and physical resources. Moreover, more crowded and complex city life increases the time pressure on the urban citizen. It has been shown that the primary source of the increased stress in everyday life is the mismatch between these demands and the individual's available resources. The European Environment Agency describes this as “urban stress” (EEA, 2020). Although some stress can be beneficial for increased alertness, athletic ability, or better focusing during an exam, it is well known that long-term urban stress can be harmful to the human body. Emotion regulation techniques such as meditation, yoga, and walking outdoors may help to reduce stress. However, urban lifestyles such as long working hours and commutes prevent the most well-known emotion regulation techniques.

Today, designers need a better understanding of the psychological and emotional impacts and threats of changing physical and social landscapes. Neuroaesthetics, which studies the neural basis of aesthetic experiences, can play a crucial role in designing public spaces that elicit positive emotional responses. Neuroarchitecture aims to create buildings that are capable of adapting to human emotions and cognitive states by modifying light and sound to influence sensory experiences (Makanadar, 2024). Incorporating natural elements such as green spaces and natural lighting can enhance mood, promoting a sense of calm and wellbeing. In contrast, exposure to unnatural light sources can adversely impact mental health, intensifying feelings of anxiety and depression. Understanding and regulating these emotional shifts can be enriched by applying neurotechnology. Consequently, two significant challenges emerge here: First, sensing stress, fatigue, anger, and other potentially harmful emotional or physical states, and second, facilitating emotion regulation in urban environments.

A third challenge relates to another pivotal component of smart cities: the sensors that can be statically deployed throughout urban areas. They enable monitoring of key parameters about the city, such as noise, air pollution, traffic levels, or availability of parking places. Sensors are mostly used as data loggers, and raw data from the nodes can be transmitted, mostly through wireless interfaces, to a server or cloud where further data processing occurs. Deploying the sensors around a city and in large quantities presents the challenge of installing a complex and costly infrastructure. In addition, questions such as how to power the devices and maintain/replace them in case of failures arise. Cities already face the challenge of replacing old infrastructures, like water distribution and sewage systems, and making the introduction of new structures is even more daunting. Thus, easy-to-deploy and easy-to-maintain sensing systems are required.

Finally, as the social economy develops, there is a rapid increase in both the global population and private automobiles. The increasing demand for mobility burdens the existing transportation infrastructure, resulting in increased road accidents, congestion, and inefficient use of energy resources, consequently causing environmental, economic, and societal impairments. Rather than building more roads, there is a need for more efficient use of currently available means of transportation through the development of intelligent transportation and logistics in addition to regulating traffic flow, throughput, and safety.

#### 3.1.2 Technologies and solutions

##### 3.1.2.1 On physiological sensing-based emotion regulation with wearables across smart cities

Since smart watches, smart wristbands, and other smart wearables such as rings have become pervasive and their sensing modalities have increased, sensing emotional states has become feasible using wearables (Can et al., 2019). There are observable physiological changes in the human body, such as a reaction to stress. Heart Rate Variation (HRV) and Electro-Dermal Activity (EDA) are well-known stress indicators. Most wearables have Photoplethysmography (PPG) sensors that can measure HRV, which changes quickly in a stressful situation. In addition, some wearables have Galvanic Skin Response sensors for detecting EDA. Although an EDA reaction is slower than an HRV response, it has been shown that multimodality improves the emotion/fatigue sensing capability (Can and Ersoy, 2023).

The ubiquity of wearables is not the only reason for the improvements in emotion sensing. The sensors create multiple streams of time series data. Recent advances in machine learning (ML) techniques, such as deep learning and faster ML hardware, have enabled more accurate models for emotion sensing. If the data is not extensive, an acceptable sensing accuracy can be achieved using classical ML techniques such as support vector machines and decision trees. With the availability of larger physiological data from wearable sensors, better overall emotion-sensing performance can be obtained using deep learning techniques. However, there are increased privacy concerns about health-related data. New techniques, such as federated learning also facilitate privacy-aware emotion sensing with better accuracy (Can and Ersoy, 2021). Then, the issue becomes acting to regulate emotions after detecting the emotional states while living in urban environments, such as working in the office or commuting. One EU-funded H2020 research project targeting these issues was Affectech: Personal Technologies for Affective Health (https://cordis.europa.eu/project/id/722022). In that project, interactive tools and wearable actuators with vibrotactile and temperature-changing elements were recommended for emotion regulation in urban environments. In that context, there is much room for improvement in urban spaces. Nudges or subtle environmental cues that encourage positive behavior can also be incorporated into the design of public spaces to promote healthy habits, such as walking or socializing. In addition, smart layouts and sound systems can further enhance the safety and accessibility of public spaces.

##### 3.1.2.2 On crowd-sensing with mobile, wearable, and iot devices across smart cities

Crowd-sourced or participatory sensing enables collecting ambient personal data, particularly from the devices carried/worn/owned by people. Smartphones, wearables, and IoT devices are the ideal platforms for sensing applications with the integrated rich set of sensors, their ubiquity, ease of use, support for mobility, and wireless interfaces (Ìncel and Özgövde, 2018). Consider the sensors available in today's smartphones: microphone, camera, GPS, light, accelerometers, gyroscope, compass, GPS, pressure, temperature, magnetic field, humidity, and heart rate, among others. The sensor set can be extended by those not integrated into the device but can be connected via wireless interfaces, such as gas and occupancy sensors. They can also communicate and complement existing, static sensing infrastructures. These devices gather ambient information and collect data about the context, such as activities, location, emotional states, and health status, of the users carrying them. Users can also be sensors by reporting their observations (Berntzen et al., 2016) through app interfaces. Programming such devices is easy, and the applications can be delivered to large populations worldwide through app stores. Hence, they enable global mobile sensor networks.

Typical applications of crowd-sourced systems in smart cities include environmental monitoring, traffic and transportation monitoring, and monitoring road conditions such as detecting road obstacles. Normally, these devices are personal, and the sensing applications are for personal purposes, such as step counters, fitness tracking, and wellbeing monitoring. However, context recognition of crowds and communities rather than individuals enables a new set of application domains in urban planning and transportation. By monitoring what is happening in urban environments, for example, it will be possible to discern those regions' typical or routine actions: which regions are used and for which activities? By analyzing common activities and transportation modes in urban settings, suitable zones and times for cycling, for instance, can be marked on maps. Alternatively, extraordinary situations can be monitored, and possible emergency or disaster situations can be detected: what if many users start running in an area where people usually sit or walk? In addition, people's transportation modes, such as cycling, car/bus, and train/metro, can be tracked, thus laying the groundwork for applications such as extracting transportation-type maps of cities.

##### 3.1.2.3 On cooperative intelligent transportation systems

In traffic, automated vehicles need to interact with other vehicles, bicycles, pedestrians, and other road users, as well as with IoT services (including via Road-Side-Units), over Dedicated Short-Range Communication or 5G-V2X networking (Zhang et al., 2023). Information exchange between Connected Automated Vehicles through Vehicle-to-Vehicle and Vehicle-to-Infrastructure wireless communication enables automated vehicles to cooperate and interact within their (socio)-cyber-physical environments. Connected automated vehicles, forming the so-called Internet of Vehicles, are predicted to transform transportation and urban life (Loke, 2019). Other automated vehicles, such as self-driving wheelchairs and self-driving motorcycles, are also being developed. In addition, cooperative Intelligent Transport Systems (e.g., cooperative driving) is an active area of research.

As the logistics industry struggles with the demand for faster, more energy-efficient, and autonomous delivery methods, the focus on last-mile delivery has intensified. Cooperative delivery mission planning, and the utilization of Unmanned Aerial Vehicles (UAVs) and Automated Ground Vehicles (AGVs) emerge as a promising alternative to conventional methods for last-mile delivery. The synchronization of UAVs with AGVs provides the advantage of delivering hard-to-reach places. These heterogeneous mobile platforms also offer a range of strategic alternatives in terms of operational time, delivery time, and fuel consumption. Moreover, since the UAVs cruise through the air, the delivery is independent of roads, traffic lights, speed limits, and pedestrian crossings. This aerial freedom is particularly beneficial in urban settings where numerous external factors can impede ground deliveries.

Meanwhile, in today's traffic, the limitations of human perception of traffic conditions and response times define the boundaries of safe inter-vehicle distances. Erroneous human driving behaviors can trigger traffic flow instabilities, which result in the so-called shockwaves. For instance, in dense traffic, an overreaction by one driver to a momentary disturbance—like a preceding vehicle's slight deceleration—can set off a ripple effect. Such disruptions might bring the traffic to a full stop, kilometers away from the original source, causing traffic jams for no apparent reason. In this respect, addressing these disturbances across the vehicle string, an aspect encapsulated by string stability, is an essential requirement for vehicle platooning. Fortunately, wireless information exchange between vehicles provides the means for overcoming sensory limitations of human or Adaptive Cruise Control (ACC) operated vehicles and, therefore, can significantly enhance the traffic flow, especially on highways.

Cooperative Adaptive Cruise Control (CACC) is an advanced system designed to regulate inter-vehicle distances, enhancing traffic flow stability and throughput. As an extension of ACCs, CACC achieves superior performance by leveraging wireless information exchange between vehicles through Dedicated Short-Range Communication (DSRC) in addition to local sensor measurements (Öncü et al., 2012, 2014). The primary aim of CACC is to pack the driving vehicles together as tightly as possible to increase traffic flow while preventing the amplification of disturbances throughout the string, known as string instability. Implementing such a system offers significant fuel savings, particularly for trucks. This is attributed to trucks' considerable frontal surface area. When these vehicles are closely packed within a platoon, aerodynamic losses are reduced, especially during highway operations (Nieuwenhuijze et al., 2012). A study by Alam et al. (2010) provides evidence for this kind of benefit: when the inter-vehicle distance between two heavy-duty trucks ranged from 3-10 m, the trailing truck saw a fuel reduction of 10–12%, while the leading truck achieved savings of 5–10%.

### 3.2 Neurotechnologies in smart cities for resilience and sustainability

#### 3.2.1 Challenges in disaster management

The World Health Organization defines disasters as any occurrence that causes loss of human life, human suffering, damage, and destruction of infrastructure and environment at a degree that requires supportive action from outside the influenced community or area (Haghani and Oh, 1996). It is possible to divide the disasters into two categories with respect to their cause: natural disasters and man-made ones. The first category is earthquakes, floods, landslides, wildfires, tsunamis, avalanches, storms, hurricanes, and pandemics. The second category includes environmental pollution, hazardous materials accidents, and terrorist attacks. Independent of the cause of the disasters, they have an expected outcome: loss of human life and economic damages. According to the report of the Centre for Research on the Epidemiology of Disasters Report (2023), 387 natural hazards and disasters occurred worldwide in 2022. They resulted in the loss of 30,704 lives, while 185 million people were affected. The corresponding economic losses amount to approximately US$ 223.4 billion. Disaster management (DM) is traditionally considered in four phases: Mitigation, preparedness, response, and recovery (Döyen et al., 2012). Mitigation involves all the activities that aim to reduce the impact of a disaster. Preparedness consists of activities to prepare the community and organizations for efficient response during a disaster. The goal of the response activities is to set up an effective relief chain to provide relief items such as food, water, medication, and shelter to affected areas. Lastly, the recovery phase aims to restore the affected people, their properties, and the neighborhood to its previous state.

Disruptions that can arise as a result of a disaster have the potential of generating a significant deterioration in the operation of critical infrastructure networks such as transportation networks, electric power distribution networks, supply chain networks, and telecommunication networks, which provide services or products to customers. For example, the shutdown of a subway station, the closure of one or more lanes on a bridge, the operation of an airport at a much-reduced capacity, and the disruption of an electric power plant may result in a significant drop in people's quality of life and wellbeing. Consequently, the concept of resilience and sustainability has emerged in the last decade as a new performance measure considered in the planning and decision-making activities related to disaster management, which formally concentrated on efficiency and effectiveness. Based on the definition of resilience in supply chains given by Ponomarov and Holcomb (2009), we can define resilience in smart cities as “the adaptive capability of the city to prepare for disruptions that occur due to unexpected (maybe sometimes expected) disasters, respond to them, and recover from them by maintaining continuity of operations at the desired level of connectedness.” Indeed, this definition aligns with the resilience of all living organisms, an innate neurobiological capacity of all living organisms, including humans. As mentioned on the website of “Neurozone^®^”, it is possible to utilize knowledge from neuroscience and brain-body system that enables humans to adapt, stay alive, and evolve. It can be helpful to establish systems to increase the resilience of smart cities in addressing the associated problems and solving them effectively (van der Walt, 2023).

#### 3.2.2 Challenges in waste management

Developing built environments that meet human needs without incurring adverse social and environmental impacts is challenging (Hamilton et al., 2002). There is, therefore, a pressing need for concerted action in maintaining a virtual cycle between human wellbeing and ecosystem health. One prominent concern in this context is urban waste. With cities growing in physical size and population, an unprecedented amount of waste is produced in urban localities. However, contemporary waste management practices face several challenges, including inadequate infrastructure, limited funding, and a lack of public consciousness regarding the importance of waste minimization and recycling. To tackle these issues, it is essential to find and implement new solutions that prioritize waste reduction, recycling, and proper disposal practices and recognize each urban environment's unique needs and characteristics.

Environmental monitoring presents its own challenges, particularly in terms of spatial coverage, time resolution, and deployment costs. Some critical indicators, such as land and water surface temperatures, ground vegetation, and alterations in watercolor, can be observed using satellite imagery, which offers extensive partial coverage. However, the frequency of measurements is sensitive to atmospheric conditions, with gaps on cloudy days. In addition, the spatial resolution of satellite imagery is currently 30 m−1 km—making it useful for regional assessments—but there is often a need for on-the-ground verification to obtain finer details.

### 3.3 Technologies and solutions

Aligned with the aims of SDG 11—to make cities inclusive, safe, resilient, and sustainable—any effort to enhance the resilience of cities to disasters and environmental risks addresses this goal. The technologies that can be used to increase resilience and sustainability in smart cities in the context of both disaster management and waste management are similar and based on devices developed with the help of information and communication technology (ICT).

A huge amount of information can be collected using ICT, including IoT-based sensors, tracking, monitoring systems, as well as monitoring systems, and crowd-sourcing mechanisms. This information—ranging from the positioning of injured people to their health status and from weather conditions to road statuses and other environmental factors—significantly aids informed decision-making across all four phases of disaster management. For example, in the preparedness phase of a hurricane, evacuation planning and strategies require decisions that maximize and ensure safety, and using accurate data, the number of people that can safely leave the affected area can be determined. In the aftermath of events like earthquakes, debris clearance, which involves the removal of debris from roads to enable vehicle movement on them to access people in need of humanitarian relief items, requires informed decisions. These decisions can only be made optimally by collecting, sharing, and using reliable and timely data related to various critical infrastructures such as buildings, roads, subways, bridges, and power and water distribution networks.

In this context, ICT utilizes various devices—RFID readers, sensors, cameras, satellites, and radars—to generate data in various forms, including structured and unstructured text, images, audio, and video. In waste management, low-cost sensors aid in automated waste segregation to increase recycling efficiency (Esmaeilian et al., 2018; Hait and Thakur, 2017) as well as monitor environmental parameters such as water quality, air quality, agriculture systems, hazardous materials, odor, and noise nuisance. Using sensors to detect hazardous substances in different ecosystems can protect human life and the environment from the detrimental impacts of these materials (Zhao et al., 2022). Gas sensors and electronic noses are used to identify odor nuisance (Jońca et al., 2022), while both level and source of noise can be detected through sensors and wireless sensor networks, enabling citizens to avoid the negative consequences of these issues (Picaut et al., 2020). The availability of such sensors supports citizen science with robust and low-cost sensor and networking technology. In addition, such networks offer improved data coverage (especially in remote or data-scarce regions). Additionally, these sensors have disaster management applications, such as assessing road debris blocking access to affected people or monitoring bridge vulnerabilities (Kaur et al., 2022).

Of course, advanced computing and IoT technologies are essential for storing, processing, and disseminating this data reliably and cost-effectively. Smart waste management integrates various IoT applications to refine waste collection, separation, data monitoring, and illegal disposal mapping. Furthermore, when paired with machine learning, IoT can also play a substantial role in coping with disasters like floods, droughts, and wildfires (Sood et al., 2018; Kaur and Sood, 2020). Sensor data, for instance, can be beneficial in keeping track of the water level in rivers in predicting floods. Overall, data, whether raw or processed using analytical techniques and machine learning algorithms, offers valuable insights for informed decision-making related to different phases of disaster management and waste management problems (Banerjee et al., 2020). Algorithms can, furthermore, be utilized for dynamic route optimization of waste collection vehicles while allowing citizens to access real-time data of waste collection processes.

In fact, utilizing ICT also contributes to real-time data monitoring and communication between all stakeholders, hence supporting decision-making in waste management as well as disaster management (Hannan et al., 2015; Banerjee et al., 2020). The integration of a myriad of technologies into waste management and disaster management can positively influence the environment and social life, thus encouraging citizens' adaptation to the smart city concept.

## 4 Looking forward: open issues for future research

In line with the scope outlined in Figure 2, this section discusses several open issues that deserve attention under three overarching and intertwined categories. These encompass (i) institutions that advocate for data-driven governance, transparency, and accountability—all crucial to support public decision-making processes in the highlighted purposive areas along with discussions on the importance of multi-stakeholder collaboration; (ii) the immediate need for initiatives such as the establishment of new educational and research programs like NeurotechEU, (iii) the implementation of regulatory frameworks and ethical guidelines.

### 4.1 Institutions for data-based governance

ICT technologies and their infrastructures are crucial means for developing smart cities. However, there is little reason to assume that human wellbeing and sustainability objectives will naturally be served by the free play of uncontrolled forces and actors' will. Given the current trajectory of the smart city agenda, one must ask: How effectively are we addressing societal needs and environmental concerns and achieving SDGs? Is there an opportunity to better align adaptive resource use and context-aware decision-making-focused research and practices with these global goals and foundational principles? What governance mechanisms can best align technology use with human wellbeing and sustainability goals in urban contexts?

In fact, the concept of crowdsensing via the devices carried/worn/owned by people for urban sensing is not novel (Lane et al., 2010). Yet, numerous considerations remain, especially regarding the effective implementation of smart city applications. In this context, from a societal and governance point of view, the role of institutional arrangements will be central when selecting, adopting, designing, implementing, and using these solutions and technologies (Ruhlandt, 2018). For instance, while offloading data processing to servers or the cloud is common, privacy concerns can hinder users from sharing their data. If possible, data can be processed in the device, but in that case, for example, a machine learning model trained for a specific device may not benefit from data collected by other devices. In this regard, emerging distributed machine learning methods, like federated learning and privacy-preserving optimization, which are gaining attention from the research community, will likely become pivotal. Hence, how can federated learning methods be effectively deployed to balance privacy concerns with efficient data processing in smart cities?

It is also worth noting that most successful machine learning techniques are supervised, relying heavily on labeled data. Most data labeling techniques expect individuals to frequently fill out standard surveys. However, the practicality of frequent survey-based data labeling in modern urban life is questionable. This pushes for a stronger emphasis on semi-supervised or unsupervised machine learning techniques, reducing the burden of ground truth collection. Note also that personal devices, typically operating on batteries, have inherent limitations. Issues like battery and power management, computational constraints, and the feasibility of running complex data processing and machine learning tasks mandate solutions. Strategies such as edge computing or task simplification might be the answer. Neuromorphic computing and in-memory computing are emerging methodologies that offer significant potential for enhancing on-device machine learning or edge-AI capabilities. Neuromorphic computing (Ivanov et al., 2022) mimics the structure and function of the human brain, enabling more energy-efficient and biologically plausible computations. In-memory computing (Ielmini and Pedretti, 2020) integrates computation and memory, reducing the need for data movement and improving performance. Recent research explores the use of these approaches. For example, Firoozi and Firoozi (2024) investigate the application of neuromorphic computing for disaster management in smart infrastructure, highlighting its potential for resilience. It seems that to achieve the envisioned benefits of a smart city, a dual approach is necessary. Designers must collect and manage local and remote sensing data and adopt a user-centered approach that prioritizes the wellbeing of individuals.

While data plays a pivotal role in shaping city governance, it can only be collected, monitored, visualized, and shared with the stakeholders. Actions can be taken according to the incoming data as a response by the governors of the cities. Therefore, it is the symbiotic relationship between technology, community, and urban infrastructure that will presumably determine the city's future trajectory. Therefore, appropriate governance mechanisms should be in place to evaluate choices, and outcomes (Vatn, 2009) and deliberate on key questions of phronetic planning (Flyvbjerg, 2001), such as: “Where are we going?”, “Is this development desirable?” and “What, if anything, should we do about it?”. This aligns with Jantsch's (1970) idea of inter-transdisciplinary university which seeks to identify and integrate values and norms as part of a policy for humanity, guiding both education and innovation, as discussed below.

### 4.2 New educational and research programs

Addressing the highlighted issues requires more than just the capabilities of ICTs. It necessitates the active involvement of other pivotal smart city components: citizens and policymakers. The complexity of sensor technologies and data transmission can be a barrier to citizen participation and citizen science, which would require training and technical support at the citizen level. Employees of government offices, municipal authorities, and NGOs also need to be trained and equipped with cutting-edge technologies that can be applied around smart cities. So, what kind of training frameworks and life-long learning activities, can be established to increase citizen engagement in smart city projects?

Moreover, urbanization is intensifying in many parts of the world, driven by rapid shifts in economic, social, political, and cultural landscapes. Today, over half of the world's population resides in urban areas, and the urban population is expected to rise to 60% by 2030 and 70% by 2050, according to estimates made by the United Nations. Therefore, there is also a pressing need to rethink the quality of life and public health in new urban developments through the lens of state-of-the-art research. Urbanization outside of Europe, particularly in its transition to metropolitization, suggests that alternative solutions and approaches are needed. Specifically, cities in regions like Africa and Asia urgently require the introduction and development of new technological capabilities and opportunities. This underlines the importance of a forward-thinking approach to urban planning and development informed by technological advancements. How can interdisciplinary educational programs be tailored to equip urban planners with neuroscience-based insights for enhancing public spaces?

In light of this, future universities are expected to develop increasingly interdisciplinary and transdisciplinary approaches to ensure that the entire education and innovation system becomes coordinated as a multi-level, multi-goal hierarchical system (Jantsch, 1970). Accordingly, transdisciplinarity should be prioritized over discipline-specific research and innovation, which often merely extends existing practices. Jantsch's (1970) framework envisions three types or levels of interdisciplinarity: technological, normative, and purposive—all interconnected in a hierarchical manner. The technological level encompasses physical technology, ranging from simple products to complex systems and their interactions with societal systems. The normative level, organized through the language of planning, focuses on directing scientific endeavors toward specific goals. Finally, the purposive level introduces values and value dynamics through interactive fields such as philosophy, ethics, arts, and religion, which structure some of the domains at the normative level in an interdisciplinary manner.

In this context, smart cities provide an excellent example of how transdisciplinarity can be effectively applied, requiring the integration of diverse fields to address complex challenges and create human wellbeing centered, sustainable environments. Integrating insights from neuroscience into urban planning aligns with Jantsch's (1970) framework, but also requires going beyond it, emphasizing the hierarchical linkage between technological, normative, and purposive interdisciplinarity in this field. Here, the first tier, technological interdisciplinarity in neurourbanism, occurs when cognitive neuroscience and psychology inform urban design to create spaces that positively influence mental health and cognitive function. For example, using neuroscience data to guide the engineering of noise-reducing and human-centered architectural features demonstrates this type of interdisciplinarity, though it alone is not sufficient. The second tier, normative interdisciplinarity, is illustrated when urban planning incorporates principles from fields like public health and environmental sciences to design spaces that actively promote wellbeing and/or sustainability. For instance, urban green spaces planned with insights from neuroscience, landscape architecture, and urban ecology foster environments that reduce stress and enhance social cohesion. The top tier, purposive interdisciplinarity, occurs when urban planning and policy intersect with deeper societal goals influenced by ethics and socio-political considerations. This involves using insights from neurourbanism to craft policies that prioritize equitable access to mental health-supportive infrastructure, aligning with ethical frameworks that emphasize social justice and inclusivity.

Each type and level of interdisciplinarity enriches urban planning by integrating knowledge across various domains. However, it is essential to always keep the top tier, the purposive level, in mind to ultimately make cities more responsive to human needs and societal aspirations. This approach ensures that urban planning moves beyond technical solutions to address deeper human and social values.

In this context, new educational and research programs such as NeurotechEU and lifelong learning for capacity building and citizen engagement in smart city projects are important for the healthy development of smart initiatives. NeurotechEU's mission, for instance, centers around leveraging neurotechnology as a conduit that provides strategic bridges between various disciplines, including neuroscience, medicine, engineering, artificial intelligence, cognitive science, robotics, social sciences, and humanities. Such endeavors thrive on fostering collaborations and synergies between experimental, theoretical, and technological groups and linking state-of-the-art multi-disciplinary research with entrepreneurial skills.

### 4.3 Addressing legal and ethical concerns

Two other key concerns that are important in smart city development are legal frameworks and ethics since the way and the extent to which they are adopted would make a difference in smart city outcomes. Moreover, in the presence of scientific and technical uncertainties, disagreements regarding the visions of smart cities and associated policies will become particularly apparent. When uncertainties and social controversies hinder consensus on factual matters, it becomes crucial to establish and protect the legitimacy of diverse viewpoints and value commitments while addressing issues effectively. Therefore, it is very important to prioritize developing laws and ethics aligned with emerging technologies and their implications. This will ensure that technological advancements are deployed in a socially responsible and sustainable way.

For instance, given that most of the devices in question are intended for personal use, prioritizing users' privacy and ensuring that using any data for large-scale applications does not violate privacy will be essential. In addition, decision-making under conflicting objectives and liability, such as autonomy vs. heteronomy, must be considered when examining automated vehicles. It is not enough to view these vehicles as autonomous entities that only sense and react to their immediate environments and goals; they should also be seen as a form of social AI that can develop cooperative awareness and behavior. To facilitate this cooperative awareness, shared perception is necessary. This can be achieved, for instance, through a shared ontology called CAM, which enables vehicles to exchange information about their immediate dynamic states, intentions, dynamic capabilities and limitations, and perceived environments. Cooperative map generation tools such as ADASIS (Ress et al., 2008) and SENSORIS (Eichberger et al., 2017) can also aid in these processes by allowing for shared data that reduces the sensing and computation requirements of individual vehicles, thereby promoting the adoption of new technologies. What other specific legal frameworks are required to protect user data privacy while facilitating shared perception?

Overall, there is consensus that by creating platforms that allow for the sharing of resources and knowledge, technology can be harnessed as a force for positive change in urban settings. However, it is also well known that excessive or irresponsible use of technology, a digital divide, or ineffective citizen participation may always compromise SDGs. Therefore, a comprehensive understanding of smartness necessitates moving beyond sole reliance on technological solutions and purposefully establishing coherent policies to improve human functionality and capabilities while respecting societal and environmental rights and ethics. How can such ethical guidelines be standardized to address liability issues in smart cities? Attention to detail will be essential, as disparities in the framing and implementation of smart city initiatives will significantly influence policy outcomes in practice.

## 5 Concluding remarks

This paper began by reviewing the current state of knowledge in the literature on using neuroscience and neurotechnological solutions in smart cities based on bibliometric analysis. It then explored some emerging themes in greater detail, mainly concerning issues with an urgent need for action in line with the SDGs, particularly human health and wellbeing as well as resilience and sustainability. Our bibliometric analysis revealed that key technologies dominate the agenda of smart city research at the intersection of neuroscience, technology, urban space, and society. However, technological arrangements and infrastructures should not be regarded as solely technical domains. There remains a need to build better synergies between societal challenges and technological solutions. Establishing direct links to the SDGs, supported by a diversified strategy fostering interdisciplinary collaboration and systemic governance, is essential. Such awareness holds significant value for academics and policymakers, providing insights into establishing disciplinary connections and areas for intervention.

Overall, the mutual constitution of technology and society requires the democratization of technology alongside shifts in governance and appraisal methods. This approach gives stakeholders a voice in how technology is regulated, governed, and used, ensuring it serves the public interest. Society must discuss generating reliable data, transforming it into actionable insights, promoting transparency, and ensuring accountability. Furthermore, the inception of new deliberative institutions seems vital to amplify public decision-making processes involving the different social groups living in the urban landscape. In sum, to navigate urban challenges effectively in tandem with the SDGs and in light of a smart city vision, it is important to acknowledge the great power of partnerships and collaboration between different parties with common interests in sustainability. While not always easy, getting different public and private actors to talk to each other and join common initiatives will be crucial.

At this point, the notion of socio-technological citizenship becomes pivotal, emphasizing the rights and responsibilities of citizens as they engage with, and are affected by, rapidly advancing technologies in urban environments. This concept highlights the role of citizens not only as users but as co-creators, decision-makers, and stakeholders in shaping and governing technology-driven initiatives. Their active involvement is essential to ensure the ethical and effective use of technological innovations that serve human wellbeing. By fostering a sense of ownership and accountability, socio-technical citizenship could bridge the gap between technological progress and societal wellbeing, promoting a more equitable and sustainable future for all. In parallel, policymakers must work toward understanding key societal challenges that smart city technologies can address. This requires deciphering the views and positions of multiple stakeholders in the community regarding these technologies and their role in The technology-society nexus. Training programs for both policymakers and citizens will surely be essential to enhance a better understanding, deployment and continuous evaluation of these technologies.

All in all, achieving true “smartness” requires a holistic and inter-transdisciplinary approach that extends beyond technology to include the proactive engagement of all stakeholders to purposefully solve urban challenges. The deployment and successful implementation of smart city initiatives depend on bridging knowledge and policy gaps and fostering an enabling environment that encourages dialogue and vibrant engagement of the broader community. This, in turn, necessitates a systems approach, addressing the underlying power structures that shape decision-making processes in the long-term.
