# Supplementary material for: Neurochallenges in smart cities: state-of-the-art, perspectives, and research directions
Source: Front Neurosci. 2024 Dec 18;18:1279668. doi: 10.3389/fnins.2024.1279668 (PMC11688368; doi:10.3389/fnins.2024.1279668)
Supplement: Supplementary file 1 [file Table_1.docx]

**Table A: List of keywords for all subject areas**

| **All Subject Areas** | | | | | | | | | |
| --- | --- | --- | --- | --- | --- | --- | --- | --- | --- |
| **Cluster 1 Keywords** | **Occurrences** | **Cluster 2 Keywords** | **Occurrences** | **Cluster 3 Keywords** | **Occurrences** | **Cluster 4 Keywords** | **Occurrences** | **Cluster 5 Keywords** | **Occurrences** |
| smart city | 9591 | cloud computing | 662 | internet of things | 4748 | blockchain | 754 | LoRa | 171 |
| sustainability | 470 | edge computing | 545 | machine learning | 2251 | security | 620 | waste management | 151 |
| information and communication technology | 337 | wireless sensor networks | 524 | deep learning | 798 | privacy preserving | 391 | LoRaWAN | 150 |
| COVID-19 | 222 | smart grid | 398 | intelligent transportation systems | 656 | cyber-physical systems | 190 | LPWAN | 121 |
| geographic information system | 199 | fog computing | 335 | artificial intelligence | 649 | cyber security | 181 |  |  |
| smart mobility | 194 | vehicular ad hoc network | 317 | sensors | 379 | authentication | 157 |  |  |
| sustainable development | 183 | 5G | 294 | convolutional neural network | 330 | smart contracts | 141 |  |  |
| urban planning | 182 | energy efficiency | 254 | artificial neural network | 274 | Industry 4.0 | 134 |  |  |
| digital twin | 172 | electric vehicles | 239 | smart home | 249 | healthcare | 125 |  |  |
| digitalization | 168 | unmanned aerial vehicle | 227 | anomaly detection | 139 |  |  |  |  |
| simulations | 158 | software defined network | 218 | long short-term memory | 139 |  |  |  |  |
| governance | 157 | optimization | 206 | object detection | 137 |  |  |  |  |
| e-government | 154 | clustering | 187 | smart parking | 135 |  |  |  |  |
| transportation | 145 | renewable energy | 178 | predictive modeling | 131 |  |  |  |  |
| mobility | 144 | smart building | 170 | classification | 124 |  |  |  |  |
| technology | 144 | crowdsensing | 151 | radio frequency identification | 117 |  |  |  |  |
| crowdsourcing | 136 | internet of vehicles | 143 | Raspberry Pi | 117 |  |  |  |  |
| autonomous vehicles | 133 | smart community | 126 | computer vision | 113 |  |  |  |  |
| urbanization | 133 | quality of service | 123 | Arduino | 110 |  |  |  |  |
| open data | 131 | energy management | 121 | air quality | 104 |  |  |  |  |
| mobile application | 127 | resource allocation | 118 | image processing | 103 |  |  |  |  |
| sustainable city | 126 | genetic algorithms | 115 | air pollution | 100 |  |  |  |  |
| innovation | 124 | reinforcement learning | 114 | support vector machine | 98 |  |  |  |  |
| building information modeling | 116 | smart meters | 108 | smart environments | 96 |  |  |  |  |
| modeling | 116 | energy consumption | 101 | global positioning system | 86 |  |  |  |  |
| smart governance | 110 | routing | 90 | intrusion detection | 86 |  |  |  |  |
| public transport | 106 | energy | 87 |  |  |  |  |  |  |
| social media | 97 |  |  |  |  |  |  |  |  |
| infrastructure | 96 |  |  |  |  |  |  |  |  |
| resilience | 92 |  |  |  |  |  |  |  |  |
| interoperability | 91 |  |  |  |  |  |  |  |  |
| ontology | 91 |  |  |  |  |  |  |  |  |
| sustainable smart city | 90 |  |  |  |  |  |  |  |  |
| architecture | 86 |  |  |  |  |  |  |  |  |

**Table B: List of keywords for computer sciences**

| **Computer Sciences** | | | | | | | | | |
| --- | --- | --- | --- | --- | --- | --- | --- | --- | --- |
| **Cluster 1 Keywords** | **Occurrences** | **Cluster 2 Keywords** | **Occurrences** | **Cluster 3 Keywords** | **Occurrences** | **Cluster 4 Keywords** | **Occurrences** | **Cluster 5 Keywords** | **Occurrences** |
| cloud computing | 581 | smart city | 6584 | internet of things | 3959 | blockchain | 645 | LoRa | 143 |
| edge computing | 501 | artificial intelligence | 518 | machine learning | 1851 | security | 542 | LoRaWAN | 133 |
| wireless sensor networks | 438 | sustainability | 225 | deep learning | 693 | privacy preserving | 339 | waste management | 120 |
| fog computing | 303 | information and communication technology | 190 | intelligent transportation systems | 533 | cyber-physical systems | 157 | LPWAN | 108 |
| vehicular ad hoc network | 283 | COVID-19 | 132 | sensors | 298 | cyber security | 155 | smart meters | 66 |
| smart grid | 265 | geographic information system | 123 | convolutional neural network | 292 | authentication | 141 |  |  |
| 5G | 257 | digital twin | 122 | artificial neural network | 235 | smart contracts | 123 |  |  |
| software defined network | 199 | crowdsourcing | 114 | smart home | 200 | healthcare | 95 |  |  |
| unmanned aerial vehicle | 186 | smart mobility | 111 | anomaly detection | 126 |  |  |  |  |
| energy efficiency | 179 | e-government | 109 | long short-term memory | 117 |  |  |  |  |
| optimization | 149 | digitalization | 101 | object detection | 117 |  |  |  |  |
| clustering | 147 | mobile application | 101 | smart parking | 112 |  |  |  |  |
| electric vehicles | 143 | open data | 100 | predictive modeling | 109 |  |  |  |  |
| crowdsensing | 136 | mobility | 99 | classification | 104 |  |  |  |  |
| internet of vehicles | 126 | smart community | 96 | computer vision | 102 |  |  |  |  |
| simulations | 126 | Industry 4.0 | 93 | Raspberry Pi | 102 |  |  |  |  |
| smart building | 120 | transportation | 92 | radio frequency identification | 101 |  |  |  |  |
| quality of service | 110 | ontology | 82 | Arduino | 94 |  |  |  |  |
| reinforcement learning | 102 | smart environments | 80 | image processing | 89 |  |  |  |  |
| resource allocation | 99 | urban planning | 78 | support vector machine | 80 |  |  |  |  |
| genetic algorithms | 96 | social networks | 77 | deep neural network | 76 |  |  |  |  |
| renewable energy | 95 | governance | 76 | intrusion detection | 75 |  |  |  |  |
| autonomous vehicles | 89 | interoperability | 74 | air quality | 70 |  |  |  |  |
| modeling | 88 | public transport | 74 | global positioning system | 70 |  |  |  |  |
| routing | 80 | natural language processing | 73 | traffic congestion | 69 |  |  |  |  |
| energy consumption | 76 | augmented reality | 72 |  |  |  |  |  |  |
| energy management | 73 | smart governance | 71 |  |  |  |  |  |  |
| multi-agent system | 70 | technology | 69 |  |  |  |  |  |  |
| wireless communication | 69 | sustainable development | 67 |  |  |  |  |  |  |
| particle swarm optimization | 68 | social media | 66 |  |  |  |  |  |  |
| architecture | 67 |  |  |  |  |  |  |  |  |
| federated learning | 67 |  |  |  |  |  |  |  |  |

**Table C: List of keywords for engineering**

| **Engineering** | | | | | | | | | |
| --- | --- | --- | --- | --- | --- | --- | --- | --- | --- |
| **Cluster 1 Keywords** | **Occurrences** | **Cluster 2 Keywords** | **Occurrences** | **Cluster 3 Keywords** | **Occurrences** | **Cluster 4 Keywords** | **Occurrences** | **Cluster 5 Keywords** | **Occurrences** |
| internet of things | 2337 | blockchain | 348 | wireless sensor networks | 286 | smart city | 3957 | artificial intelligence | 319 |
| machine learning | 1010 | security | 312 | smart grid | 219 | sustainability | 185 | cyber-physical systems | 97 |
| deep learning | 355 | cloud computing | 308 | electric vehicles | 144 | information and communication technology | 140 | cyber security | 85 |
| intelligent transportation systems | 316 | edge computing | 247 | energy efficiency | 141 | smart mobility | 84 | digital twin | 74 |
| sensors | 222 | privacy preserving | 176 | optimization | 114 | sustainable development | 74 | simulations | 71 |
| convolutional neural network | 150 | vehicular ad hoc network | 166 | renewable energy | 100 | transportation | 70 | autonomous vehicles | 69 |
| smart home | 129 | fog computing | 159 | LoRa | 98 | COVİD-19 | 66 | Industry 4.0 | 69 |
| artificial neural network | 127 | 5G | 150 | smart building | 97 | digitalization | 62 | building information modeling | 66 |
| predictive modeling | 75 | unmanned aerial vehicle | 126 | energy management | 78 | healthcare | 58 | geographic information system | 63 |
| waste management | 73 | software defined network | 90 | LoRaWAN | 77 | sustainable city | 56 | modeling | 52 |
| long short-term memory | 72 | clustering | 77 | LPWAN | 76 | urban planning | 55 | augmented reality | 45 |
| object detection | 72 | authentication | 73 | smart meters | 73 | public transport | 51 | crowdsourcing | 43 |
| radio frequency identification | 72 | internet of vehicles | 68 | smart community | 63 | technology | 51 |  |  |
| smart parking | 71 | crowdsensing | 65 | microgrid | 58 | interoperability | 47 |  |  |
| Raspberry Pi | 65 | quality of service | 65 | energy consumption | 56 | urbanization | 46 |  |  |
| Arduino | 58 | resource allocation | 60 | genetic algorithms | 54 | mobility | 45 |  |  |
| classification | 55 | smart contracts | 58 | reinforcement learning | 54 | sustainable smart city | 45 |  |  |
| image processing | 54 | wireless communication | 47 | energy | 46 | e-government | 41 |  |  |
| computer vision | 52 | architecture | 44 | particle swarm optimization | 44 | resilience | 41 |  |  |
| anomaly detection | 51 | application | 43 | energy harvesting | 43 |  |  |  |  |
| mobile application | 51 | reliability | 43 | demand response | 41 |  |  |  |  |
| intrusion detection | 49 | drones | 41 |  |  |  |  |  |  |
| support vector machine | 48 |  |  |  |  |  |  |  |  |
| global positioning system | 46 |  |  |  |  |  |  |  |  |
| air pollution | 43 |  |  |  |  |  |  |  |  |
| deep neural network | 43 |  |  |  |  |  |  |  |  |

**Table D: List of keywords for social sciences**

| **Social Sciences** | | | | | | | | | |
| --- | --- | --- | --- | --- | --- | --- | --- | --- | --- |
| **Cluster 1 Keywords** | **Occurrences** | **Cluster 2 Keywords** | **Occurrences** | **Cluster 3 Keywords** | **Occurrences** | **Cluster 4 Keywords** | **Occurrences** | **Cluster 5 Keywords** | **Occurrences** |
| internet of things | 575 | smart city | 2364 | intelligent transportation systems | 112 | geographic information system | 72 | social media | 36 |
| machine learning | 498 | sustainability | 191 | smart mobility | 58 | open data | 44 | smart tourism | 28 |
| blockchain | 125 | information and communication technology | 130 | sensors | 54 | building information modeling | 41 | natural language processing | 21 |
| artificial intelligence | 116 | urban planning | 95 | urbanization | 51 | crowdsourcing | 38 |  |  |
| deep learning | 110 | COVID-19 | 87 | innovation | 50 | virtual reality | 27 |  |  |
| cloud computing | 82 | governance | 84 | city | 48 | augmented reality | 23 |  |  |
| privacy preserving | 81 | sustainable city | 68 | transportation | 47 | crowdsensing | 23 |  |  |
| security | 80 | sustainable development | 66 | mobility | 42 | smart campus | 23 |  |  |
| smart grid | 69 | e-government | 62 | infrastructure | 38 | data visualization | 22 |  |  |
| wireless sensor networks | 68 | digitalization | 55 | autonomous vehicles | 36 | ontology | 21 |  |  |
| edge computing | 57 | technology | 53 | planning | 33 |  |  |  |  |
| smart home | 53 | sustainable urban development | 52 | public transport | 32 |  |  |  |  |
| convolutional neural network | 48 | smart governance | 46 | case studies | 31 |  |  |  |  |
| artificial neural network | 47 | urban governance | 44 | modeling | 29 |  |  |  |  |
| 5G | 42 | citizen participation | 42 | decision making | 25 |  |  |  |  |
| sustainable smart city | 40 | urban development | 42 | management | 24 |  |  |  |  |
| unmanned aerial vehicle | 40 | resilience | 35 | mobile application | 24 |  |  |  |  |
| electric vehicles | 39 | India | 31 | visualization | 24 |  |  |  |  |
| energy efficiency | 38 | China | 29 | urban mobility | 23 |  |  |  |  |
| clustering | 36 | digital twin | 28 | radio frequency identification | 22 |  |  |  |  |
| cyber security | 36 | climate change | 27 | bibliometric analysis | 21 |  |  |  |  |
| renewable energy | 35 | pandemic | 27 | network | 21 |  |  |  |  |
| optimization | 34 | smart community | 27 | simulations | 21 |  |  |  |  |
| cyber-physical systems | 32 | smart urbanism | 27 |  |  |  |  |  |  |
| fog computing | 32 | participation | 26 |  |  |  |  |  |  |
| software defined network | 31 | local government | 24 |  |  |  |  |  |  |
| smart building | 30 | quality of life | 24 |  |  |  |  |  |  |
| predictive modeling | 27 | urban policy | 23 |  |  |  |  |  |  |
| classification | 26 |  |  |  |  |  |  |  |  |
| long short-term memory | 25 |  |  |  |  |  |  |  |  |
| Industry 4.0 | 24 |  |  |  |  |  |  |  |  |
| object detection | 23 |  |  |  |  |  |  |  |  |
| particle swarm optimization | 23 |  |  |  |  |  |  |  |  |
| smart contracts | 22 |  |  |  |  |  |  |  |  |
| architecture | 21 |  |  |  |  |  |  |  |  |
| smart environments | 21 |  |  |  |  |  |  |  |  |

**Table E: List of keywords for environmental sciences**

| **Environmental Sciences** | | | | | | | | | |
| --- | --- | --- | --- | --- | --- | --- | --- | --- | --- |
| **Cluster 1 Keywords** | **Occurrences** | **Cluster 2 Keywords** | **Occurrences** | **Cluster 3 Keywords** | **Occurrences** | **Cluster 4 Keywords** | **Occurrences** | **Cluster 5 Keywords** | **Occurrences** |
| smart city | 862 | sustainability | 113 | internet of things | 183 | digitalization | 25 | renewable energy | 28 |
| information and communication technology | 53 | urban planning | 42 | machine learning | 118 | smart mobility | 22 | smart grid | 28 |
| sustainable development | 47 | artificial intelligence | 40 | intelligent transportation systems | 35 | urban development | 18 | blockchain | 26 |
| sustainable city | 45 | governance | 28 | cloud computing | 29 | security | 16 | electric vehicles | 21 |
| COVID-19 | 32 | geographic information system | 27 | sensors | 23 | clustering | 12 | wireless sensor networks | 16 |
| innovation | 27 | technology | 25 | deep learning | 22 | energy | 12 | smart home | 14 |
| sustainable smart city | 27 | waste management | 22 | artificial neural network | 13 | Industry 4.0 | 12 | 5G | 12 |
| sustainable urban development | 24 | infrastructure | 21 | arduino | 12 | sustainable transport | 10 | e-government | 10 |
| city | 22 | transportation | 19 | image processing | 12 | 3D city model | 9 | energy management | 9 |
| urbanization | 21 | building information modeling | 17 | convolutional neural network | 11 | privacy preserving | 9 | cyber security | 8 |
| climate change | 20 | energy efficiency | 15 | global positioning system | 11 | environment | 8 | microgrid | 8 |
| circular economy | 19 | optimization | 15 | data visualization | 10 | visualization | 8 |  |  |
| resilience | 18 | bibliometric analysis | 13 | LoRa | 9 |  |  |  |  |
| smart governance | 17 | planning | 13 | unmanned aerial vehicle | 9 |  |  |  |  |
| air pollution | 16 | built environment | 12 | global system for mobile communication | 8 |  |  |  |  |
| air quality | 15 | mobility | 12 | long short-term memory | 8 |  |  |  |  |
| sharing economy | 13 | smart building | 12 | smart campus | 8 |  |  |  |  |
| urban governance | 13 | sustainable development goals | 12 | smart meters | 8 |  |  |  |  |
| India | 11 | case studies | 11 |  |  |  |  |  |  |
| mobile application | 11 | quality of life | 11 |  |  |  |  |  |  |
| urban transformation | 11 | public space | 10 |  |  |  |  |  |  |
| pandemic | 10 | smart urbanism | 10 |  |  |  |  |  |  |
| smart technologies | 10 | decision making | 9 |  |  |  |  |  |  |
| smart tourism | 10 | indicators | 9 |  |  |  |  |  |  |
| eco-city | 9 | policy | 9 |  |  |  |  |  |  |
| social innovation | 9 | simulations | 9 |  |  |  |  |  |  |
| social media | 9 | accessibility | 8 |  |  |  |  |  |  |
| energy transition | 8 | local government | 8 |  |  |  |  |  |  |
| remote sensing | 8 |  |  |  |  |  |  |  |  |
| urban heat island | 8 |  |  |  |  |  |  |  |  |
| urban policy | 8 |  |  |  |  |  |  |  |  |
